# Complex Coacervate Emulsions as a Strategy to Stabilize Enzymes for Catalysis in Organic Solvents

**DOI:** 10.1021/acsmacrolett.5c00708

**Published:** 2025-12-24

**Authors:** Jussara Alves Penido, Stephanie P. Le, Adhithi Varadarajan, S. Thayumanavan, Sarah L. Perry, Watson Loh

**Affiliations:** † Instituto de Química, 28132 Universidade Estadual de Campinas, Campinas, SP Brazil, 13083-970; ‡ Department of Chemical and Biomolecular Engineering, 14707 University of Massachusetts Amherst, Amherst, Massachusetts 01003, United States; § Department of Chemistry, University of Massachusetts Amherst, Amherst, Massachusetts 01003, United States; ∥ Department of Biomedical Engineering, University of Massachusetts Amherst, Amherst, Massachusetts 01003, United States

## Abstract

Complex coacervates have emerged as versatile platforms for protein encapsulation, enabling enzymatic catalysis in aqueous environments. Despite their potential, applications of coacervates are limited by the substrate solubility in water. In this study, we present a protocol to stabilize enzyme-loaded coacervate droplets in water-immiscible organic solvents via the formation of highly stable emulsions. These emulsions were formed using coacervates composed of poly­(diallyldimethylammonium hydroxide) and poly­(acrylic acid), stabilized by a polystyrene-based, amphiphilic, anionic copolymer in toluene, chlorobenzene, chloroform, and dichloromethane. The resulting microdroplets display exceptional resistance to coalescence, including after centrifugation, and remain stable for weeks. This stability facilitates their separation and redispersion for use in repeated catalytic applications. Using α-chymotrypsin as a model enzyme, we show that the aqueous microenvironment within the droplets maintains enzyme stability over time and enables biocatalysis in nonaqueous media.

## Graphical abstract

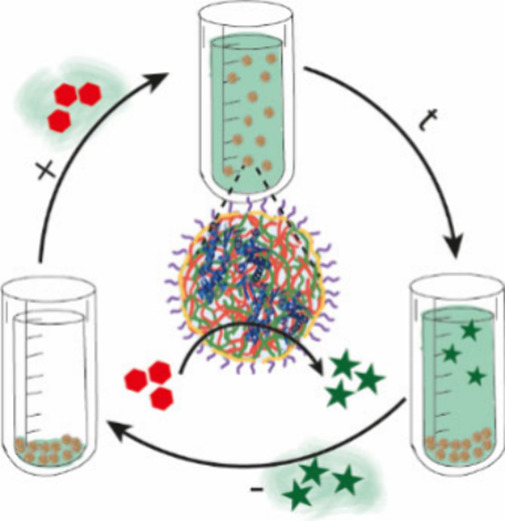

Complex coacervates have been widely explored for the encapsulation and stabilization of proteins, offering a unique environment for enzymatic catalysis in aqueous media.^1–4^ Formed through the associative liquid–liquid phase separation of oppositely charged polyelectrolytes, coacervates provide a dense, polymer-rich matrix that can enhance enzyme stability by protecting against denaturation and aggregation.^4–9^ Moreover, coacervation can regulate enzymatic activity by modulating the partitioning of substrates and products, thereby shifting reaction equilibria.^3,10^ Consequently, coacervate-based systems have been successfully employed in various aqueous-phase biocatalysis applications, including hydrolysis,^11–14^ biomolecule synthesis and transcription,^15–17^ oxidation–reduction processes,^18–21^ cascade reactions,^22–25^ and protein expression.^26,27^ Coacervate microdroplets can also act as membrane-free biomimetic protocells capable of supporting multiple simultaneous reactions,^22,24,28,29^ which some hypotheses suggest played a role in the emergence of life.^30–33^

One challenge in utilizing coacervates is their tendency to coalesce. Consequently, various authors have endeavored to stabilize these droplets in aqueous environments to prevent coalescence.^34^ One strategy involves employing copolymers with neutral and charged monomers.^34–38^ These copolymers concentrate at the interface, introducing steric repulsion among microdroplets and imparting long-term stability to the dispersions. Another approach involves cross-linking the coacervate to prevent coalescence, though this can potentially limit the transport of molecules into or out of the droplet.^31,39^ A further alternative relies on Pickering-type stabilization.^40–44^ These strategies have each demonstrated efficacy in stabilizing the droplets for extended periods, showcasing considerable promise for applications in aqueous systems.

Beyond droplet stabilization, another challenge involves the application of coacervate-based systems to reactions involving hydrophobic substrates. Despite considerable progress, most studies have utilized aqueous-phase biocatalysis.^12,14,18,23^ However, many industrially relevant enzymatic processes involve hydrophobic substrates that exhibit limited or no solubility in water.^45,46^ Performing such reactions in organic solvents introduces new obstacles, particularly regarding enzyme stability.^47,48^ Given the well-established role of coacervates in stabilizing enzymes in aqueous media, their extension as protective compartments for biocatalysis in organic solvents presents a compelling opportunity. Recent findings have shown that coacervate phases can persist in organic environments,^49,50^ thereby broadening the potential for enzyme-mediated transformations in nonaqueous systems. For instance, complex coacervate core micelles (C3Ms) formed from poly­(oligo­(ethylene glycol) methacrylate)-*b*-poly­(4-vinyl *N*-methylpyridyl iodide and poly­(acrylic acid) were robust in polar organic solvent mixtures, such as ethanol and dimethyl methylphosphonate, enabling the dispersion and stabilization of organophosphate hydrolase enzymes in these media.^51^

Although the use of coacervates in organic solvents has been demonstrated with C3Ms, a major drawback of these nanoscale assemblies is the challenge of recovering them for reuse. Moreover, C3Ms have not yet been reported in water-immiscible organic solvents, further emphasizing the need for alternative strategies. In this context, coacervates that are stable in organic media and can be easily recovered offer a promising route toward efficient and recyclable biocatalysis in nonaqueous environments.

Here, we propose a framework for stabilizing coacervate droplets to enable enzymatic catalysis in organic solvents by creating an aqueous microdomain that is stabilizing for enzymes. To evaluate our approach, we prepared coacervates of poly­(diallyldimethylammonium hydroxide) and poly­(acrylic acid), referred to as PDADMA-PA, in a series of different organic solvents, stabilized by a polystyrene-based amphiphilic anionic random copolymer (Figure 1a). We encapsulated α-chymotrypsin within these coacervates and evaluated its catalytic activity using a fluorogenic peptide substrate (Figure 1b). Additionally, we investigated the stability of the emulsion, the enzymatic activity, and the recyclability of the coacervate droplets to assess their potential for repeated catalytic cycles. These findings demonstrate the potential of coacervate-based emulsions as versatile platforms for biocatalysis in organic solvents, expanding the scope of coacervate-mediated enzymatic reactions beyond aqueous environments.

**Figure 1:**
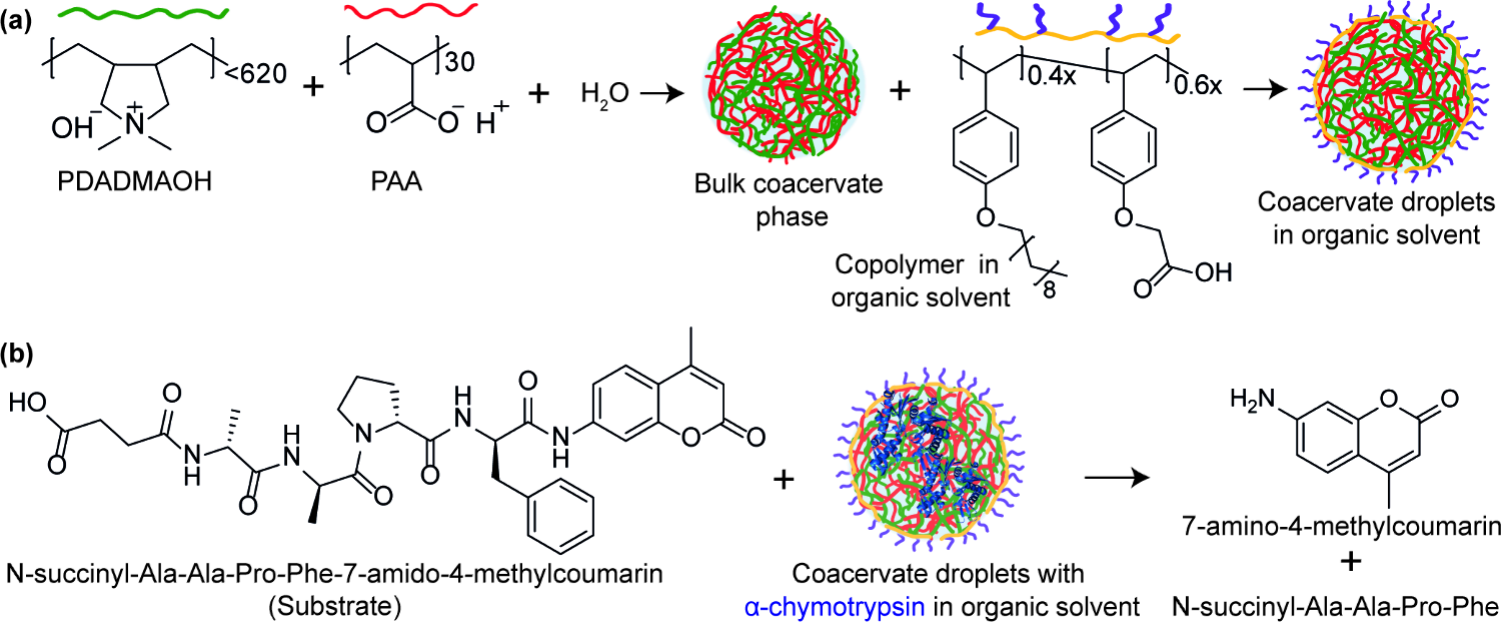
(a) Schematic representation of the formation of stabilized complex coacervate emulsions. Coacervates form by mixing aqueous poly­(diallyldimethylammonium hydroxide) (PDADMAOH) and poly­(acrylic acid) (PAA) at charge equivalence, followed by dispersion with a polystyrene-based amphiphilic anionic random copolymer in organic solvent. (b) Schematic of coacervate encapsulating α-chymotrypsin to hydrolyze N-succinyl-Ala-Ala-Pro-Phe-7-amido-4-methylcoumarin to produce a fluorescent product.

Initial attempts to generate an emulsion by directly dispersing the coacervate phase in organic solvents at a concentration of 10 mg/mL without stabilizer were unsuccessful. Even after vigorous vortexing and bath sonication, the coacervate persisted as a single bulk phase and failed to break into droplets. Given that stable emulsions were not achievable under those conditions, we shifted our strategy to designing a copolymer that could preferentially localize at the coacervate-solvent interface, thereby stabilizing the droplets.^35,37^ Therefore, we synthesized a polystyrene-based amphiphilic anionic random copolymer containing both hydrophilic and hydrophobic functionalities to stabilize coacervate-in-oil emulsions (see Supporting Information). This copolymer has been previously shown to self-assemble into spherical nanometric aggregates in organic solvents, with water confined within the structure.^52^

Incorporation of the copolymer enabled emulsification: at 10 mg/mL of coacervate and 0.4 mg/mL of copolymer in toluene, vortexing for 10 min resulted in a turbid suspension containing spherical liquid droplets with an average diameter of 2.6 ± 0.8 μm (Figure 2a,b). Over an hour, the turbidity decreased as a white sediment formed at the bottom of the vessel (Figure 2a,c). Remarkably, these droplets did not coalesce and could easily be resuspended with gentle stirring. Their size distribution remained stable both immediately after preparation, after redispersion, and after 24 h (Figure 2b). Even after 2 weeks, the droplets remained small, with an average size of 4.8 ± 2.0 μm (Figure S1).

**Figure 2:**
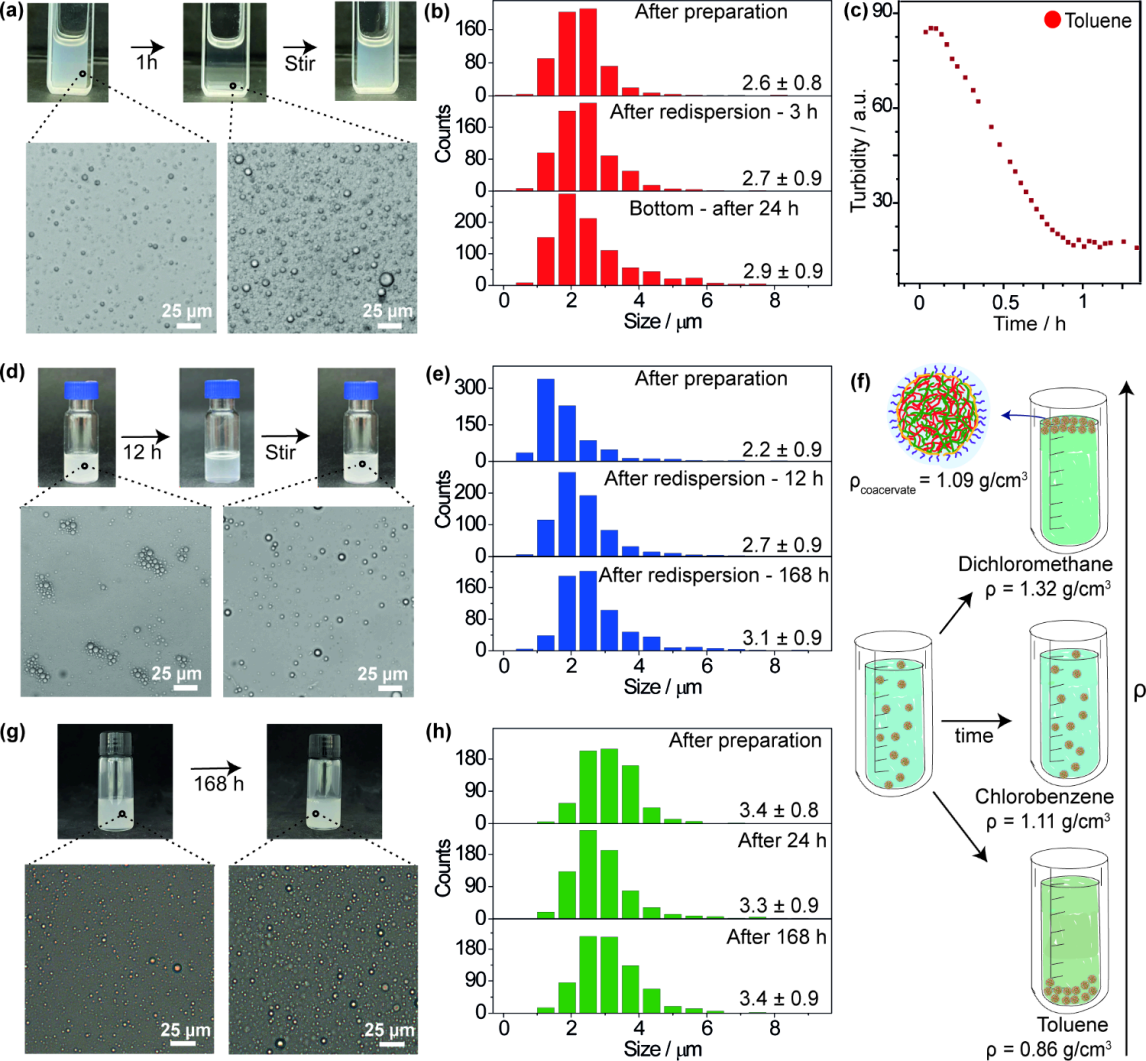
(a, d, g) Photographs and optical micrographs of the stabilized coacervate emulsions in (a) toluene, (d) dichloromethane, and (g) chlorobenzene over time. (b, e, h) Droplet size distributions for the systems in (a), (d), and (g) measured immediately after preparation, after a second agitation, and after an extended period. (c) Turbidity vs time for the system in toluene, showing droplet sedimentation. (f) Schematic of coacervate emulsions in organic solvents with different densities.

The remarkable stability of this system likely stems from the strategic location of the copolymer at the coacervate–organic solvent interface. The random copolymer allows for multiple interaction points along the coacervate interface, with anionic comonomers interacting with the coacervate phase, while hydrophobic monomers extend into the organic solvent. This amphiphilic configuration avoids full integration of the copolymer into the coacervate phase, thereby stabilizing the droplets and preventing their coalescence over time.^35^

Stable emulsions of coacervate droplets were similarly obtained in dichloromethane and chlorobenzene (Figure 2d,g). In both systems, the average droplet sizes remained small and did not change significantly over the course of 1 week (Figures 2e,h). Despite the lower polarity and dipole moment of toluene compared to dichloromethane or chlorobenzene, the copolymer effectively prevented droplet coalescence in all systems, demonstrating its versatility in stabilizing emulsions across different solvent environments.

One distinction between these systems is the density difference between the solvents and the coacervate. Because the coacervate has a density of 1.09 g/cm^3^ at 25 °C,^53^ higher than toluene and smaller than dichloromethane, the coacervate sediments in toluene and floats in dichloromethane (Figure 2f). Interestingly, due to the close match between the density of chlorobenzene (1.11 g/cm^3^) and the coacervate phase, the droplets remained suspended in the medium, resulting in persistent turbidity over time (Figure 2g). This density matching facilitates the long-term stability of the emulsion but would require alternative methods than sedimentation to separate and recover the droplets.

We also tested whether the coacervate droplets could withstand centrifugation to facilitate faster separation than is achievable via sedimentation. We prepared the coacervate dispersion in toluene and subjected it to centrifugation at 47 × g for 5 min, repeating the process three times. After centrifugation, a white layer consisting of discrete droplets formed at the bottom of the container and it was easily redispersed with gentle agitation (Figure 3). The average droplet size increased from 2.6 ± 0.8 μm before centrifugation to 4.0 ± 1.0 μm after the third cycle (Figure 3a). Nevertheless, the droplets remained small, and easily redispersible, demonstrating the efficiency of the copolymer in preventing coalescence and highlighting the potential for multiple reuse cycles, even under forced sedimentation.

**Figure 3:**
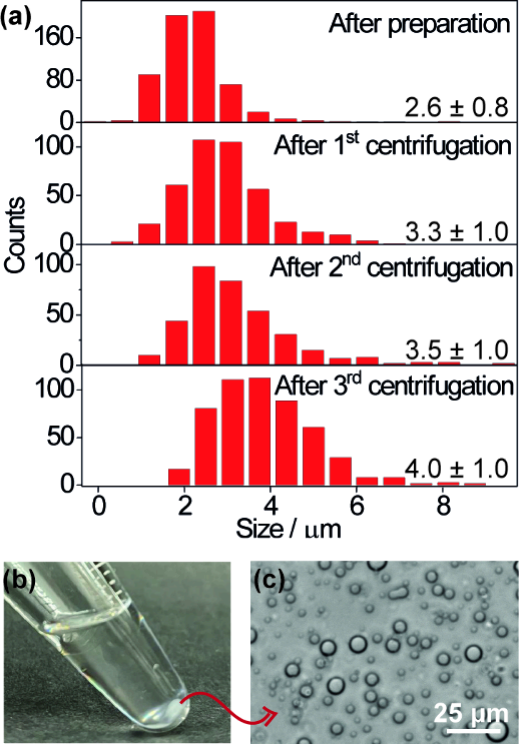
(a) Droplet size distribution after preparation, and following the first, second, and third centrifugation. (b) Photograph of the stabilized coacervate emulsions in toluene after centrifugation, showing a white coacervate layer at the bottom of the tube. (c) Optical micrograph of the sedimented droplets after centrifugation.

To evaluate the potential of coacervates to stabilize enzymes for use in organic solvents, we selected α-chymotrypsin as a model enzyme. Coacervates were prepared, lyophilized, and subsequently rehydrated with a solution containing fluorescein isothiocyanate-labeled α-chymotrypsin (FITC-α-chymotrypsin). These coacervates were then used to prepare emulsions in a copolymer solution of either toluene or dichloromethane. Confocal microscopy (Figure 4a) revealed that the enzyme was preferentially concentrated and homogeneously distributed within the coacervate droplets and did not alter the size of the coacervate droplets when compared to enzyme-free systems (Figure S2). The enzyme was strongly localized in the coacervate phase, with <0.04% detected in toluene after centrifugation and <0.08% in dichloromethane (Figure S3). While the enzyme was not expected to partition into the organic phase, we hypothesize that the measured signal was a result of small enzyme-copolymer aggregates or coacervate nanodroplets that were not fully sedimented during centrifugation.

**Figure 4:**
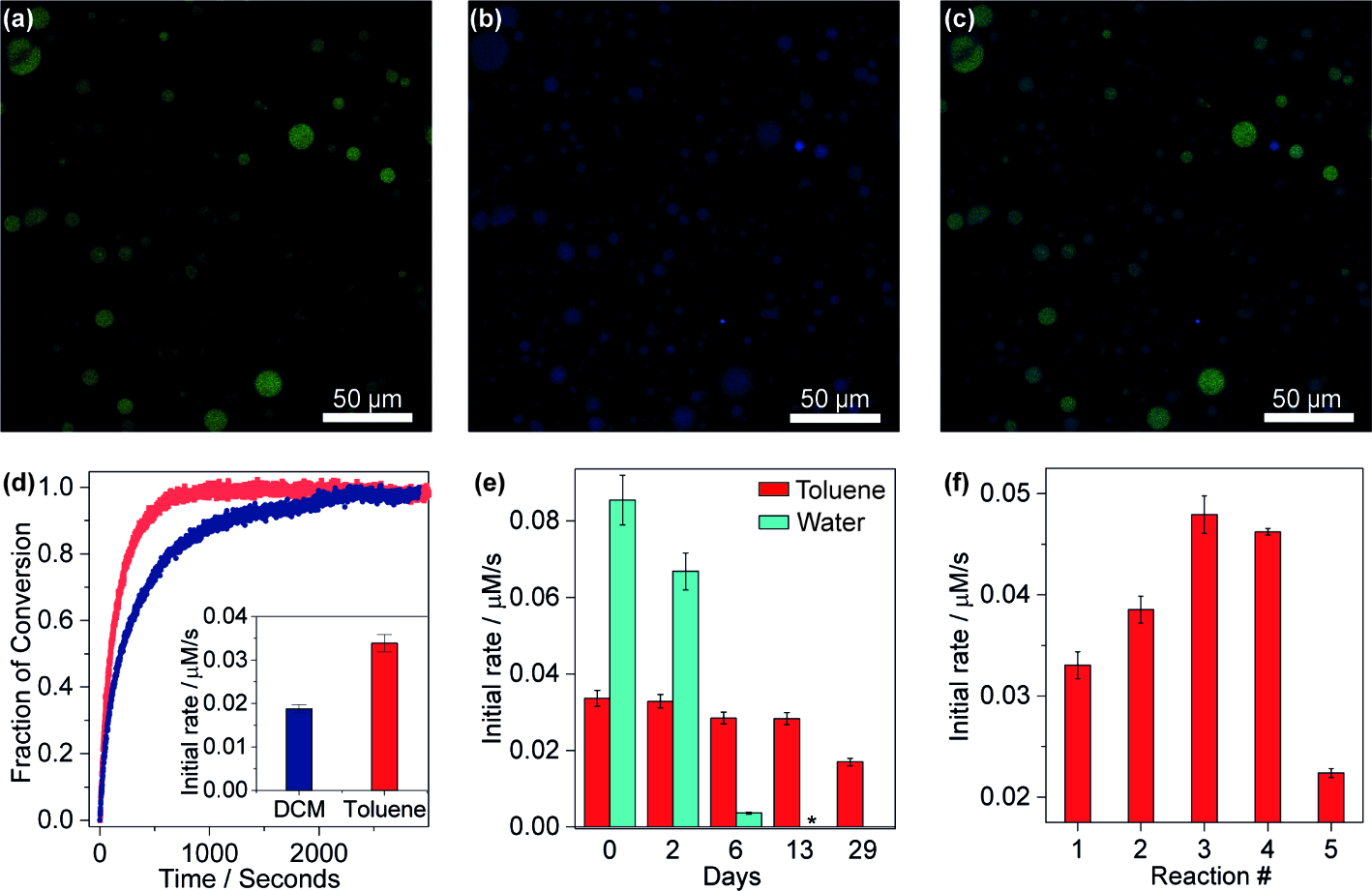
Confocal fluorescence micrographs of coacervate droplets in toluene with (a) FITC-labeled α-chymotrypsin, (b) the fluorescent product 7-amino-4-methylcoumarin, and (c) the merged image showing enzyme and product colocalization. (d) Fraction of conversion over time for stabilized coacervates with 0.22 μM α-chymotrypsin in toluene and dichloromethane after substrate addition. (e) Initial reaction rates in coacervates in toluene over time, compared to free enzyme in aqueous solution (pH 8.9). (f) Initial reaction rates over five cycles using the same stabilized coacervate droplets in toluene, replacing the solvent between cycles. The fraction of conversion vs time for (e) and (f) is shown in Figures S6 and S7.

Subsequently, the activity of α-chymotrypsin within the coacervate droplets was assessed by monitoring the hydrolysis of the fluorogenic substrate. Confocal imaging showed that the fluorescent product concentrates inside the coacervate droplets (Figures 4b and S2). Monitoring the fluorescence intensity of the reaction product over time showed complete conversion using emulsions in both toluene and dichloromethane (Figure 4d). This indicates that the coacervate preserved the catalytic activity of α-chymotrypsin, despite the enzyme being both insoluble and inactive in solvent alone (Figure S4). We would also note that the droplet size increased slightly after the reaction (Figure S2), consistent with a buildup of material.

We determined the initial reaction rate by analyzing the slope in the early linear region (up to 20% conversion, where the substrate remains in excess). For free enzyme in water, this rate was 0.085 ± 0.002 μmol/s, whereas in coacervates in toluene it was approximately 2.5-fold lower (Figure 4e). To decouple solvent effects from those of the coacervate microenvironment, we measured the rate for enzyme encapsulated in coacervate dispersed in water; the initial rate was similar to that in the toluene emulsion (Figure S5), suggesting that it is the coacervate environment itself, rather than the presence of an organic solvent, that suppresses the catalytic rate.^54^ This attenuation is consistent with prior reports that polycations can interfere with chymotrypsin activity,^55,56^ an effect that could potentially be minimized in the future through polymer design.

Remarkably, when we measured the initial rates over a period of 13 days using aliquots from the same batch of encapsulated enzyme at room temperature (25 °C), the values for the system in toluene remained nearly constant, with only modest decreases detected (Figure 4e). In contrast, the enzyme free in aqueous solution exhibited a progressive loss of activity, reaching a significantly lower initial rate after 7 days (Figure 4e). This finding confirms that, in addition to facilitating enzyme catalysis in organic solvent, the coacervate also enhances enzyme stability over time. This stabilization likely arises from the protective microenvironment provided by the coacervate matrix, which limits enzyme autoproteolysis, aggregation, and exposure to denaturing conditions. Similar stabilization effects have also been reported for biomolecules in fully aqueous coacervates, where macromolecular crowding and nonspecific interactions with the coacervate matrix favor the folded and functional states of proteins.^3–6^

In addition to stability over time, we also explored the potential for product inhibition by performing sequential additions of substrate after the reaction ran to completion. At a low initial substrate concentration (1.5 μM), the reaction rate remained unchanged across five consecutive additions (Figure S8a,b). However, when the substrate concentration was increased to (4.1 μM), a progressive decline in the initial reaction rate was observed (Figure S8c,d), suggesting product accumulation inhibits the enzyme, as has been seen in reverse micelles.^57^

In the context of employing coacervate-based emulsions as enzymatic microreactors, product inhibition can be overcome by washing the product out of the coacervate droplets. Thus, we evaluated droplet recyclability. In each reaction cycle, fresh substrate was introduced, and the reaction was run to completion before centrifuging to isolate the droplets. The supernatant, containing approximately 50% of the fluorescent reaction product, was then removed, and a fresh solvent phase was added (see Supporting Information). Remarkably, the coacervate droplets maintained their catalytic activity, sustaining a consistent reaction rate over the first four cycles (Figure 4f). A slight increase in reaction rate was observed up to the third cycle, followed by a progressive decrease from the fourth to the fifth cycle. These trends likely reflect the balance of three potential factors. One factor that may account for the initial rate increase is the reduction in water content of the coacervate phase during washing with toluene. In contrast, (1) loss of enzyme into toluene phase during solvent exchange, and (2) loss of coacervate droplets during each washing step could contribute to the decrease in rate. While the direct loss of enzyme should be low due to strong partitioning, loss of coacervate becomes more significant over time as the coacervate reservoir becomes progressively smaller. Thus, the relative loss of the same quantity of droplets during washing could lead to a disproportionately large reduction in catalytic activity in the later cycles. Considering enzyme partition coefficients and the volume ratio between the phases, we estimated an enzyme loss of ca. 3.25% each cycle. This loss could potentially be mitigated through copolymer optimization and/or modification of the coacervate matrix to further enhance enzyme retention, or via the addition of additional enzyme. Overall, these results underscore the potential of coacervate droplets as highly efficient and recyclable enzymatic microreactors, offering a viable strategy for enzyme recovery and reuse. At the same time, they identify water and enzyme retention, and droplet recovery as key aspects for future study and improvement.

To broaden the applicability of our protocol, we evaluated the ability to form coacervate-in-organic solvent emulsions and maintain enzyme activity in a range of solvents with different polarities and water miscibilities (Table 1). Overall, two factors emerged as critical for identifying a useful catalytic system: (1) the presence and sufficient solubility of the stabilizing copolymer in the solvent and (2) the immiscibility of the solvent and water. Without copolymer, emulsions did not form in any of the tested solvents. In hexane, where copolymer solubility is very low, emulsions could be formed by vortexing but were only transiently stable. In addition to the solvents already mentioned, we also formed emulsions in chloroform. By contrast, highly water-miscible solvents such as diethyl ether, ethyl acetate, and ethanol caused rapid solidification of the coacervate phase, due to water extraction from the droplets.

**Table 1:** Solubility Parameters and Dielectric Constants of Selected Organic Solvents, Along with Observations on the Stability of Coacervate-Based Emulsions

solvent	dielectric constant	solubility of OS in water^58^ (mg/mL)	solubility of water in OS^58^ (mg/mL)	emulsion stability (10 mg/mL of coacervate)
hexane	1.88	0.14	0.10	fast droplet coalescence
toluene	2.38	0.52	0.33	stable, reusable
chlorobenzene	5.62	0.5	0.4	stable, reusable
chloroform	4.81	8.15	0.56	stable
dichloromethane	8.93	16.0	2.4	stable
diethyl ether	4.33	68.9	12.6	solid precipitation
ethyl acetate	6.02	87	33	solid precipitation
ethanol	24.3	∞	∞	solid precipitation

Enzymatic reactions proceeded in all coacervate-in-organic solvent emulsions tested (Figure S9), however both the initial rate and the long-term enzyme stability varied depending on the solvent. In toluene and chlorobenzene, activity was largely preserved for at least 13 days, outperforming the free enzyme in aqueous solution under the same storage conditions (Figures 4e and S6). Both systems also supported multiple catalytic cycles (Figures 4f and S10). However, in more water-miscible solvents such as chloroform and dichloromethane, prolonged incubation revealed a time-dependent decrease in enzymatic activity, reaching a minimum after 7 days (Figure S11), likely due to slow denaturation due to the solvent in the coacervate. In hexane, catalytic activity was detectable but was low, most likely due to limited substrate solubility. These results emphasize that solvent selection – balancing copolymer solubility, water immiscibility, and substrate compatibility – is key to designing robust coacervate-based catalytic systems.

The results of this study demonstrate the formation of highly stable coacervate-in-organic solvent emulsions capable of encapsulating enzymes and supporting efficient biocatalysis in nonaqueous environments. These emulsions provide an effective medium to overcome limitations for biocatalysis with water-insoluble substrates and significantly enhance enzyme stability over extended periods compared to aqueous solutions. Also, since the coacervate emulsion can be formed in solvents with different polarities, it can be used for substrates with different polarities. Moreover, these emulsions are stable enough to endure multiple use cycles, maintaining enzymatic activity and allowing for enzyme recovery.

Looking forward, we have identified requirements for the solubility of the stabilizing copolymer in the organic solvent, and that the solvent should not significantly partition into the coacervate phase. With these requirements in mind, it will be interesting to explore what types of applications are enabled by this coacervate platform. Additionally, once a system of interest is identified, further optimization of the coacervating polymers could help to further enhance the reaction rate.

## Supplementary Material



## Notes

Materials and methods; Supplementary figures: coacervate emulsion and enzymatic reaction (S1 to S11) and characterization of the copolymers (S12 to S16); Partition coefficient and product distribution analysis (PDF)

## Abbreviations

C3Ms: complex coacervate core micelles
PDADMAOH: poly­(diallyldimethylammonium hydroxide)
PAA: poly­(acrylic acid)
