# Supplementary material for: Complex Coacervate Emulsions as a Strategy to Stabilize Enzymes for Catalysis in Organic Solvents
Source: ACS Macro Lett. 2025 Dec 24;15(1):143–50. doi: 10.1021/acsmacrolett.5c00708 (PMC12825365; doi:10.1021/acsmacrolett.5c00708)
Supplement: Supplementary file 1 [file mz5c00708_si_001.pdf]

Supporting Information

Complex Coacervate Emulsions as a Strategy to Stabilize Enzymes for Catalysis in Organic Solvents

Jussara Alves Penido,[1,2] Stephanie P. Le,[3] Adhithi Varadarajan,[2] S. Thayumanavan,[3,4] Sarah L. Perry,[2*] and Watson Loh[1*]

1. Instituto de Química, Universidade Estadual de Campinas, Campinas, SP, Brazil, 13083-970.

2. Department of Chemical and Biomolecular Engineering, University of Massachusetts Amherst, Amherst, MA, USA, 01003.

3. Department of Chemistry, University of Massachusetts Amherst, Amherst, MA, USA, 01003.

4. Department of Biomedical Engineering, University of Massachusetts Amherst, Amherst, MA, USA 01003.

*Corresponding authors e-mail address: perrys@engin.umass.edu (S.L. Perry) and wloh@unicamp.br (W. Loh)

Table of Contents

Materials and Methods 1

Rationale and Preliminary Tests for Copolymer Structure and Emulsification Conditions 8

Supplementary Figures 9

Effect of Water-Saturated Organic Solvents on Coacervate Dispersion 20

Partition Coefficient and Product Distribution Analysis 21

Quantification of Copolymer Remaining in the Continuous Phase 22

Characterization of the Copolymers 24

References 28

Materials and Methods

Materials

All chemicals, 4-cyano-4-(phenylcarbonothioylthio)pentanoic acid (chain transfer agent), dithiothreitol (DTT), fluorescein-NHS (fluorescein N-hydroxysuccinimide ester), α-chymotrypsin (enzyme) from bovine pancreas, N-succinyl-Ala-Ala-Pro-Phe-7-amido-4-methylcoumarin (substrate), poly(diallyldimethylammonium chloride) (PDADMAC; nominal M_n < 100,000 g mol^{-1}), and poly(acrylic acid) (PAA; nominal M_n = 2,000 g mol^{-1}) were used as received unless otherwise noted. 2,2′-Azobis(2-methylpropionitrile) (AIBN) was purified by recrystallization prior to use. All reagents were obtained from Sigma-Aldrich or Fisher Scientific. Dowex® Monosphere® 550A (OH) type ion

exchange resin was purchased from Sigma-Aldrich. Polystyrene monomers 1 and 2 were synthesized following previously reported procedures.[1,2]

Synthesis of the Copolymers P0 and P1

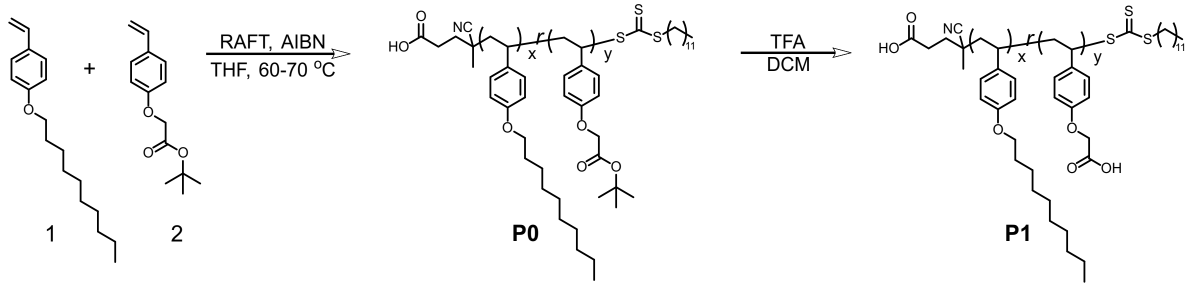

Scheme S1. Synthesis of random copolymers **P0** and **P1**. **P0** was obtained via RAFT polymerization of monomers **1** and **2** in THF at 60-70°C using 4-cyano-4-[(dodecylsulfanylthiocarbonyl)sulfanyl]pentanoic acid as RAFT agent and AIBN as initiator. Subsequent deprotection of the tert-butyl groups in **P0** with trifluoroacetic acid in DCM yielded **P1**. The detailed synthetic procedure is provided in the text below.

Synthesis of Random Copolymer P0:

To a Schlenk tube, compound **1** (148 mg, 0.569 mmol, 40 eq), compound **2** (200 mg 0.854 mmol, 60 eq), 4-cyano-4-[(dodecylsulfanylthiocarbonyl)sulfanyl] pentanoic acid (RAFT agent, 5.74 mg, 0.0142 mmol, 1 eq) and 2,2′-azobis-(2-methylpropionitrile) (AIBN, 0.466 mg, 0.00284 mmol, 0.2 eq) were dissolved in 400 μL dry tetrahydrofuran (THF). Note: AIBN was added from a concentrated stock solution dissolved in THF. The mixture was degassed by four cycles freeze/thaw cycles, sealed under argon, and heated at 65°C under argon for 12 hrs. The reaction was stopped by inserting the tube into cold water and then opening in the air. Afterwards the polymer was dialyzed against a 7:3 mixture by volume of dichloromethane (DCM) and methanol for one day with a molecular weight cut-off 3,500 g/mol (Spectrum Laboratories Inc.). The solution was dried under vacuum to yield 230 mg (66% yield) of **P0** as a pale-yellow solid polymer. From ^{1}H NMR, integration of methylene proton next to the phenol in both alkyl unit (c) and carboxylate unit (g) provided the molar ratio of monomers to be 4:6 (decyl/carboxylate). Molecular weight assessed by gel permeation chromatography (GPC) in THF: M_n: 9.3 kDa, Đ:1.03 (Figure S14). ^{1}H NMR (400 MHz, $CDCl_3$) δ 6.58-6.31, 4.43, 3.86, 1.75, 1.57, 1.47, 1.28, 0.89 (Figure S13). ^{13}C NMR (100 MHz, $CDCl_3$) δ 168.21, 157.04, 155.77, 128.29, 113.99, 81.92, 67.89, 65.82, 39.38, 31.91, 29.63, 29.36, 28.06, 26.19, 22.69, 14.13 (Figure S16).

Deprotection of Random Copolymer P1:

To a 25 mL vial, 230 mg of **P0** was dissolved in 1.6 mL of DCM and 0.6 mL of trifluoroacetic acid was added to the mixture and stirred overnight to remove the tert-butyl protecting groups. The mixture was then dialyzed against a 6:4 mixture of DCM and methanol for one day with a molecular weight cut-off of 3,500 g/mol (Spectrum Laboratories Inc.). The solution was dried, producing 176 mg of **P1** as a solid yellow polymer. From ^{1}H NMR, a sharp decrease in integration at δ 1.44 suggested the successful deprotection of tert-butyl group. From ^{1}H NMR, integration of proton (c) and (g) again confirmed the molar ratio of monomers to be 4:6 (decyl/carboxylate). ^{1}H NMR (400 MHz, $CDCl_3$) δ 6.59, 4.85, 4.65, 3.88, 3.80, 3.49, 1.76, 1.44, 1.28, 0.88 (Figure S17). ^{13}C NMR (100 MHz, $CDCl_3$) δ 155.52, 155.50, 128.35, 114.33, 67.93, 65.44, 65.02, 39.42, 31.91, 29.61, 29.35, 26.13, 22.69, 14.13 (Figure S16).

Characterization of the Copolymers:

^{1}H-NMR and ^{13}CNMR spectra were recorded on a 400 MHz spectrometer in $CDCl_3$, using residual proton resonance of the solvents as internal standard. Chemical shifts are reported in parts per million (ppm). Ratios between hydrophobic and hydrophilic units were calculated based on the characteristic peaks of the monomers in ^{1}H NMR. The corresponding spectra are shown in Figures S15 to S18.

GPC was used to estimate the molecular weight of polymers using THF as eluent and 1 μL of toluene was added as the internal reference. Polystyrene standards were used for calibration and data analysis, and the resulting molecular weight distributions are presented in Figure S14.

Coacervate Preparation by the Complex Salt Method:

Coacervates were prepared using the complex salt approach,[3,4] which enables the formation of polyelectrolyte complexes without the presence of small counterions. This method involves the titration of a cationic homopolymer solution in its hydroxide form (PDADMAOH), obtained by passing PDADMAC through Dowex® Monosphere® 550A (OH) type strong-base anion exchange resin (total exchange capacity ≥ 1.1 eq/L). Ion exchange was performed by stirring the polymer solution with 30 g of resin for 2 h, transferring it to 30 g of fresh resin for an additional 1 h, and repeating this step once more (total exchange time: 4 h). The resulting PDADMAOH solution was titrated into a solution of the anionic homopolymer poly(acrylic acid) (PAA), with both polymer solutions prepared at a concentration of 40 mM. Titration was carried out up to the equivalence point (8.9 ± 0.4), as determined by charge

balance. Following titration, the resulting mixture was left to equilibrate for 24 h, centrifuged for 2 h to promote macroscopic phase separation, and stored in a thermostated bath at 25.0 ± 0.1°C.

Coacervate-Organic Solvent Emulsion Preparation:

To generate the emulsion, the coacervate phase was separated from the dilute phase by centrifugation at 47×g for 30 min at 25 °C (Eppendorf centrifuge, 5804R) and then dispersed in an organic solution of an amphiphilic anionic random copolymer based on polystyrene (0.4 mg/mL). For emulsions in organic solvents, all steps were performed in glass centrifuge tubes to avoid solvent-plastic interactions. The mixture was vortexed for 10 minutes, yielding a final coacervate concentration of 10 mg/mL.

Coacervate Preparation with Enzyme and Enzymatic Activity:

α-chymotrypsin was selected as a model enzyme to evaluate the potential of coacervates to stabilize enzymes for use in organic solvents (Table S1). The experimentally reported pI for this protein lies in the range of 8.75-9.1,[5–7] while the theoretical value calculated using MATLAB script[8] is 9.7 (Table S1). Therefore, at the coacervate pH used (8.9), the enzyme is expected to carry a very low net charge. To allow for the addition of a known amount of enzyme, the coacervate phase was separated as described above, transferred to pre-weighed microtubes, and the wet coacervate was accurately weighed. The volume of the hydrated coacervate was calculated using its density (1.09 g/cm^3). The coacervate was then lyophilized to remove water and quantify its solid content and then rehydrated with an aqueous solution of α-chymotrypsin (1 mg/mL). To promote thorough mixing and complete rehydration of the coacervate-enzyme system, the mixture underwent four centrifugation cycles of 15 minutes each. After every cycle, the tube was inverted to redistribute the contents and then centrifuged again in the new orientation. Following this process, the final enzyme concentration within the coacervate was 0.557 mg/mL (22.3 µM), calculated from the amount of enzyme added and the volume of the hydrated coacervate, and the hydrated coacervate contained 55.7% water by weight.

Table S1. Calculated pI, positive and negative charges, and net charge of α-chymotrypsin at pH 8.9.

pI	Positive charges	Negative charges	Net charge
9.70	19.37	17.00	2.37

The pI and charge calculations were performed with a MATLAB script, as previously reported by Blocher McTigue et al.[9]

To assess enzymatic activity, solution of the fluorogenic substrate N-succinyl-Ala-Ala-Pro-Phe-7-amido-4-methylcoumarin was prepared in organic solvent at a concentration of 0.03 mM. This solution was added to the coacervate–organic solvent emulsion containing α-chymotrypsin at an overall concentration of 0.223 μM (22.3 μM in the coacervate), to achieve a final substrate concentration of either 1.5 or 4.2 μM, corresponding to approximately 7- and 19-fold excess over the enzyme concentration, respectively.

The formation of the fluorescent product via enzymatic hydrolysis was monitored in real time using a photon-counting spectrofluorometer (PC1, ISS Inc., USA), with excitation at 365 nm and emission at 403 nm. Measurements were collected every 3 seconds, and data acquisition continued until a plateau in fluorescence intensity was observed, indicating complete conversion of the substrate to the fluorescent product. Fluorescence values were then normalized to this maximum signal (set as a conversion fraction of 1), with all other readings expressed as fractions of this value, corresponding to the extent of substrate conversion at each time point. Control experiments were performed using free α-chymotrypsin (0.223 μM) in either aqueous buffer or in the corresponding organic solvent, maintaining the same enzyme concentration used in the coacervate–organic solvent emulsions.

In addition, we investigated the effect of incubation time on enzymatic activity in the organic phase. For this, fresh coacervate-enzyme emulsions in organic solvent were prepared and stored under identical conditions. Enzymatic activity was assessed after 0, 2, 6, 13, and 30 days of incubation. For each time point, a new (previously unused) sample was used to ensure that the measured activity reflected only the stability of the enzyme over time, not cumulative effects from prior reactions.

Reuse and Recycle of the Coacervate Droplets for Enzymatic Catalysis:

To investigate the potential for reusing and recycling coacervate droplets in enzymatic catalysis, experiments were conducted using the same concentrations of enzyme (0.223 μM), coacervate, and substrate solution as described previously. In both protocols, a defined volume of the fluorogenic substrate solution (prepared at 0.03 mM in organic solvent) was added to the emulsion to reach the desired final concentration in the system.

In the successive substrate addition experiments, the substrate was added to reach a final concentration of 1.5 or 4.0 μM in the reaction mixture. Once the reaction was complete, as indicated by a plateau in fluorescence intensity, a new aliquot of substrate solution was added to return the sample to the desired substrate concentration. This process was repeated for five consecutive cycles, without altering the coacervate droplets between additions, to evaluate whether the system could sustain multiple rounds of catalysis under continuous use.

In the recycling experiments, the substrate was added to achieve a final concentration of 4.0 μM. After the reaction reached completion, the emulsion was centrifuged using glass Falcon tubes, and the supernatant (containing some of the reaction product) was carefully decanted. A fresh solution of the amphiphilic copolymer in toluene was then added to the coacervate phase. The system was vortexed for 30 seconds to redisperse the coacervate droplets in the emulsion, and a new aliquot of substrate solution was added to start the next reaction cycle. This recycling process was repeated for five cycles, with enzymatic activity monitored in each cycle by measuring the increase in fluorescence over time.

Fluorescent Labelling of α-Chymotrypsin.

Chymotrypsin was fluorescently labeled using fluorescein N-hydroxysuccinimide ester (fluorescein-NHS), which selectively reacts with primary amine groups (typically found on lysine residues and the N-terminus of proteins) under mildly basic conditions. The enzyme solution was first prepared at a concentration of 10 mg/mL in carbonate buffer (100 mM, pH 8.5), which provides the optimal pH for NHS ester coupling. Separately, fluorescein-NHS was dissolved in dimethyl sulfoxide (DMSO) at a concentration of 1 mg/mL. Then, 50 μL of the dye solution was added dropwise to 2.5 mL of the enzyme solution under stirring. The reaction mixture was allowed to stir overnight at room temperature in the dark to prevent photobleaching and to maximize labeling efficiency.

To remove unreacted dye and other small molecules, the reaction mixture was dialyzed against Milli-Q water for five days with frequent water changes, using a membrane with a molecular weight cut off (MWCO) of 3.5 kDa regenerated cellulose membrane (Spectra/Por™ 3, Spectrum Laboratories, USA). After dialysis, the labeled protein solution was lyophilized, and the resulting dry powder was stored at -20 °C, protected from light. In subsequent experiments, the labeled enzyme was used at 0.1 – 1% (w/w) relative to the total protein concentration. The exact amount was optimized based on fluorescence signal intensity in each assay.

Quantification of Enzyme in the Organic Phase.

The loss of enzyme to the continuous phase in the coacervate-in-organic-solvent emulsions was estimated using FITC-labeled chymotrypsin. Emulsions were prepared as described in Section *Coacervate Preparation with Enzyme* for both toluene and dichloromethane systems. The samples were centrifuged at 47 × g for 30 min at 25 °C (Eppendorf 5804R) to separate the coacervate droplets from the supernatant. The fluorescence intensity of the resulting supernatant was measured at 525 nm (I_{525}). These values (Figures S3a,c) were compared with the corresponding calibration curves (Figures S3b,d)

to convert fluorescence intensity into enzyme concentration. The resulting concentrations were then used to determine the amount of enzyme present in the organic phase.

The calibration curve was constructed by titrating increasing volumes of enzyme-loaded coacervate suspension into pure organic solvent (toluene or dichloromethane), yielding samples with progressively higher coacervate content while taking solvent effects into account. Fluorescence emission at 525 nm was recorded for each sample, and the resulting data were used to establish the correlation between I_{525} and enzyme concentration under these predominantly aqueous microenvironment conditions (Figures S3b,d).

Emulsion Characterization:

Optical Microscopy:

Coacervate droplets in the coacervate–organic solvent emulsions were visualized in brightfield mode using an Axio Observer Z1 inverted microscope (Carl Zeiss Microscopy LLC) equipped with 20× and 40× objectives. For sample preparation, 20 µL of the emulsion was deposited onto a glass microscope slide and covered with a coverslip. To minimize solvent evaporation, the coverslip was sealed to the slide using epoxy resin. Droplet diameters were quantified by image analysis of at least 500 droplets across three images.

Confocal Fluorescence Microscopy:

Coacervate droplets in the coacervate–organic solvent emulsions containing FITC-labeled chymotrypsin and the fluorescent enzymatic product (7-amino-4-methylcoumarin) were imaged in confocal mode using a Zeiss LSM780 Upright microscope (Zeiss Microscopy) equipped with a 40×/1.3 NA oil immersion objective. Excitation was performed using a 405 nm laser for 7-amino-4-methylcoumarin and a 488 nm laser for FITC. Emission was collected in the 430–480 nm range for 7-amino-4-methylcoumarin and in the 500–550 nm range for FITC. Control samples containing only one of the fluorescent species were analyzed to assess potential cross-excitation. These tests confirmed selective excitation and emission of each fluorophore, with negligible spectral overlap under the conditions used.

Rationale and Preliminary Tests for Copolymer Structure and Emulsification Conditions

To identify an amphiphilic polymer capable of stabilizing coacervate droplets in organic solvents, we initially evaluated several block copolymers, including poly(acrylic acid)-*b*-poly(ethylene oxide) (PAA_{33}-*b*-PEO_{45}), PAA_{72}-*b*-PEO_{136}, and poly(oligo(ethylene glycol) methacrylate)-*b*-poly(acrylic acid) ($POEGMA_{50}$-*b*-PAA_{58}). Because the PEO block is soluble in both dichloromethane and chloroform,[10] we hypothesized that these block copolymers could form a stabilizing shell around the coacervate droplets, with the PEO block extending into the organic phase. However, none of the block copolymers tested were effective at stabilizing the coacervate in either toluene, dichloromethane or chloroform.

In contrast, the polystyrene-based amphiphilic anionic random copolymer containing both hydrophilic and hydrophobic functionalities used in this study provided efficient stabilization. Its structure allows for multiple interaction points distributed along the chain, enabling the anionic comonomers to interact with the coacervate phase while the hydrophobic monomers extend into the organic solvent. This interfacial anchoring mechanism is consistent with previous observations in aqueous systems, where random copolymers have also been shown to effectively stabilize coacervate droplets.[11,12]

To determine the appropriate ratio of coacervate to copolymer, we performed preliminary emulsification tests by keeping the coacervate concentration constant at 10 mg/mL while varying the copolymer concentration (0.15, 0.4, 0.8 and 1.4 mg/mL). At 0.15 mg/mL, the resulting droplets were larger, indicating insufficient interfacial coverage. Increasing the copolymer concentration to 0.4 and 0.8 mg/mL produced smaller and more stable droplet; however, there was no significant difference between these two higher concentrations. At 1.4 mg/mL the droplets were even smaller. We selected 0.4 mg/mL as the copolymer concentration for all subsequent experiments, as it provided efficient emulsification while still enabling easy separation of the coacervate droplets from the continuous phase.

Supplementary Figures

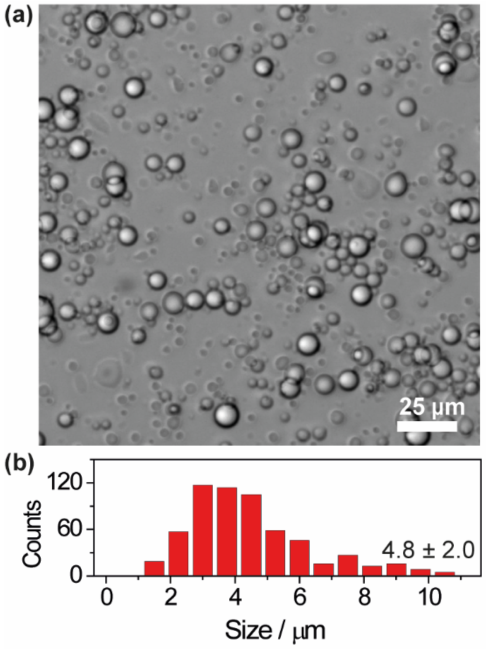

Figure S1. Characterization of the emulsion formed by the coacervate (10 mg/mL) in toluene with copolymer (0.4 mg/mL). **(a)** Optical micrograph of the droplets at the bottom after two weeks of preparation. **(b)** Size distribution of the settled droplets after two weeks.

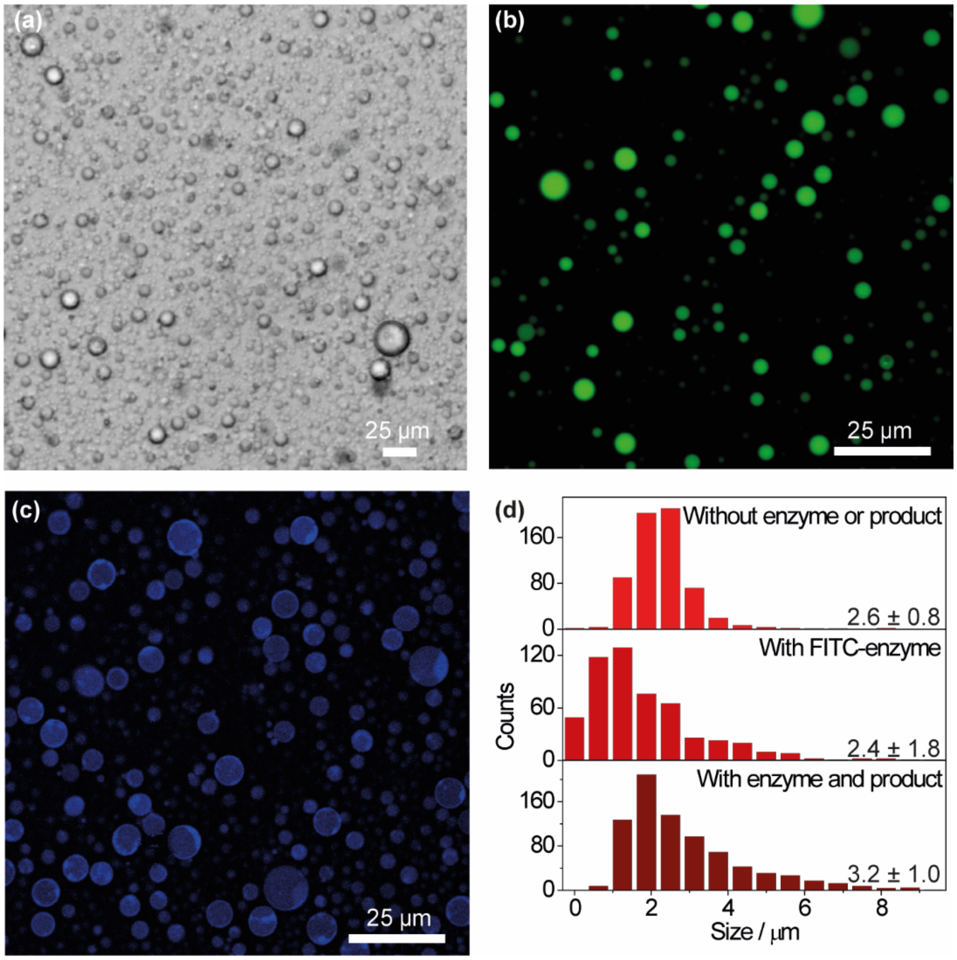

Figure S2. Characterization of the emulsion formed by coacervates (10 mg/mL) in toluene with copolymer (0.4 mg/mL) after preparation. **(a)** Optical micrograph of the system without enzyme or product. **(b)** Confocal fluorescence micrograph of the system with FITC-labeled α-chymotrypsin at 5 µg/mL, and **(c)** confocal fluorescence micrograph of the system with the enzymatic product 7-amino-4-methylcoumarin (AMC) at 4.2 µM. **(d)** Droplet size distribution, based on optical and confocal micrographs.

Statistical analysis (Figure S2d) revealed that the droplets with enzyme and product (average size of 3.2 ± 1.0 µm) were significantly larger than those without enzyme or product (2.6 ± 0.8 µm) or those with enzyme only (2.4 ± 1.8 µm), while no significant difference was observed between the latter two populations. Nevertheless, although the difference is statistically significant, the absolute change in droplet diameter is relatively small (<1 µm), indicating that overall droplet size distributions remain comparable across all conditions.

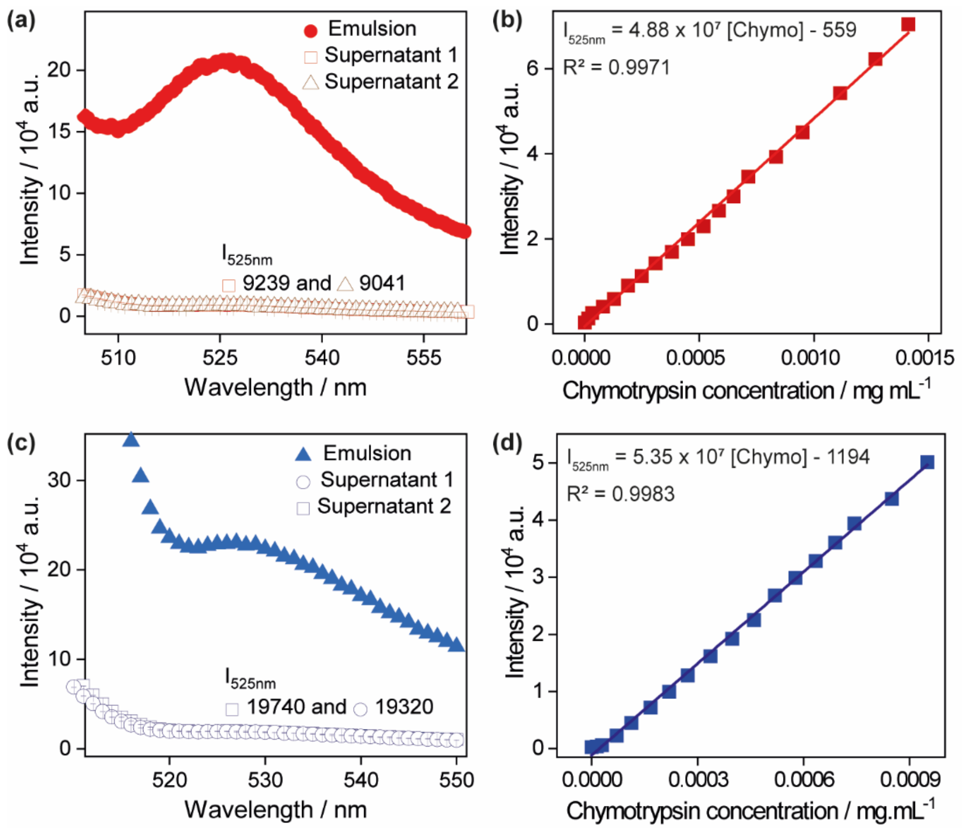

Figure S3. Partitioning behavior of FITC-labeled chymotrypsin in coacervate-based systems dispersed in organic solvents. Fluorescence emission spectra of the emulsion and corresponding supernatant (after droplet removal) in **(a)** toluene and **(c)** dichloromethane, acquired with excitation at 495 nm and emission at 525 nm. Calibration curves for FITC-chymotrypsin quantification in **(b)** toluene and **(d)** dichloromethane, respectively, used to determine the apparent partition coefficient of the enzyme between the coacervate droplets and the organic phase. The solid lines correspond to the linear regression fits used to calculate the calibration equations (shown in the figure).

The I_{525} values in Figures S3a,c correspond to the measured fluorescence intensities of FITC-chymotrypsin in the supernatant (after centrifugation and decanting) for the systems in toluene and dichloromethane, respectively. These values were then compared with the calibration curves (Figures S3b,d), which establish the correlation between I_{525} and enzyme concentration in the supernatant. This correlation enabled us to convert the measured intensities into concentrations and, in turn, to quantify the loss of enzyme to the organic phase.

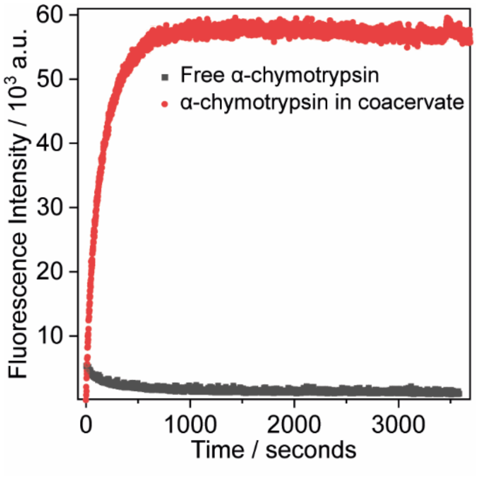

Figure S4. Fluorescence measurements over time for α-chymotrypsin (5 µg/mL) (■) and α-chymotrypsin (5 µg/mL) (●) in coacervate (10 mg/mL) in toluene following the addition of the substrate N-succinyl-Ala-Ala-Pro-Phe-7-amido-4-methylcoumarin. No significant product formation was observed, indicating negligible enzymatic activity in the absence of coacervates.

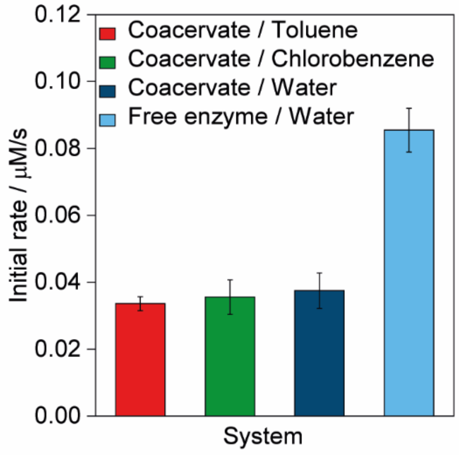

Figure S5. Initial reaction rates of α-chymotrypsin (5 µg/mL) in coacervate-based emulsions (10 mg/mL) and copolymer (0.4 mg/mL) in (■) toluene, (■) chlorobenzene, (■) water and (■) free chymotrypsin (5 µg/mL) in aqueous solution (pH 8.9), following substrate addition.

The initial reaction rates of α-chymotrypsin in the different coacervate-based emulsions (Figure S5) were not statistically different from each other, but all were significantly lower than the rate of free enzyme in aqueous solution.

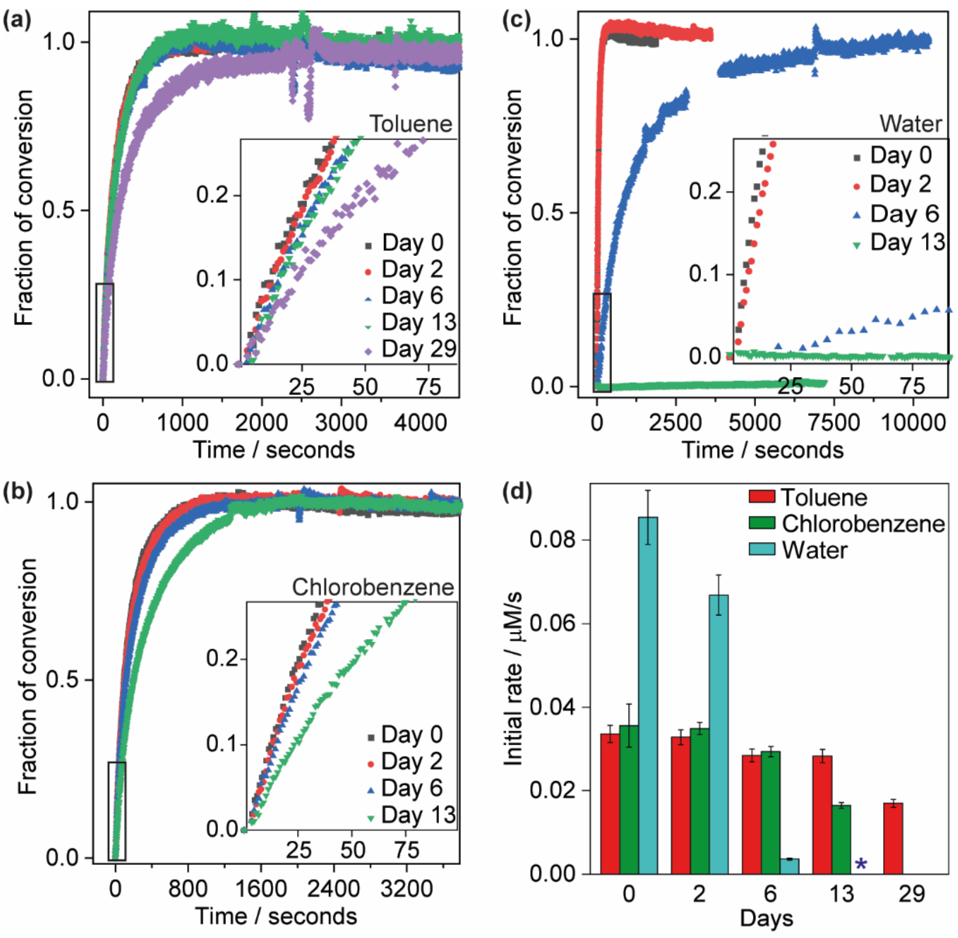

Figure S6. Fluorescence measurements over time for coacervate-based emulsions (10 mg/mL) containing chymotrypsin (5 µg/mL) and copolymer (0.4 mg/mL) in **(a)** toluene and **(b)** chlorobenzene, and **(c)** free chymotrypsin (5 µg/mL) in aqueous solution (pH 8.9), following substrate addition. Enzymatic activity was evaluated after (■) 0, (●) 2, (▲) 6, (▼) 13, and (♦) 29 days of storage, demonstrating the effect of encapsulation on long-term enzyme stability. **(d)** Initial reaction rates of α-chymotrypsin in coacervate/toluene and coacervate/chlorobenzene over time, compared to free enzyme in aqueous solution (pH 8.9). The asterisk (*) for chymotrypsin in water indicates that the initial rate of the enzyme in water was very low (below 6.8×10^{-6} µM/s). Day 0 corresponds to the measurement performed immediately after sample preparation.

As shown in Figure S6d, for emulsions in toluene, enzyme activity remained stable up to day 2. A statistically significant decrease was first detected at day 6, reaching its lowest value by day 29. For emulsions in chlorobenzene, enzyme activity was unchanged up to day 2. A significant decrease was first observed at day 6 and became most pronounced by day 13. In water, activity declined even faster, with a significant drop occurring by day 2 and near-complete loss by day 13.

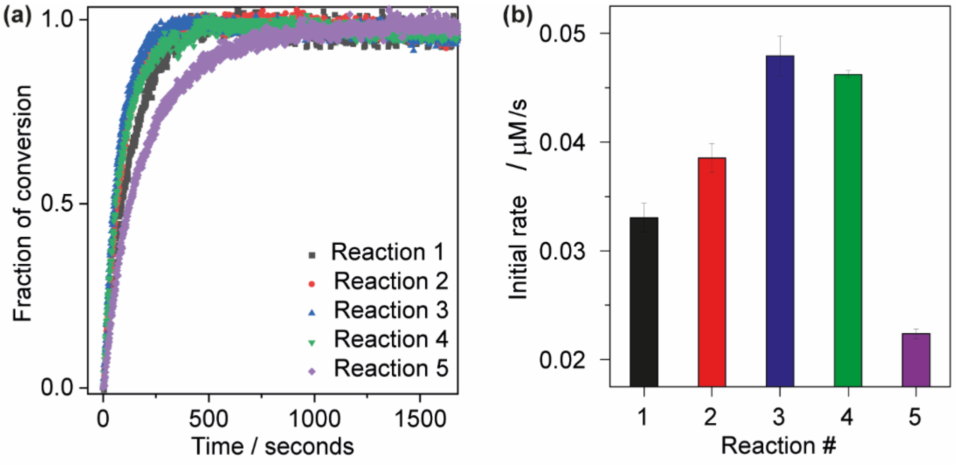

Figure S7. (a) Fluorescence measurements over time for coacervate-based emulsions (10 mg/mL) containing chymotrypsin (5 µg/mL) and copolymer (0.4 mg/mL) in toluene following substrate addition over five cycles using the same coacervate droplets. Enzymatic activity was evaluated after (■) 1, (●) 2, (▲) 3, (▼) 4, and (♦) 5 cycles of reactions, demonstrating the efficient reuse without significant loss of enzymatic activity. **(b)** The corresponding initial reaction rates extracted from each cycle.

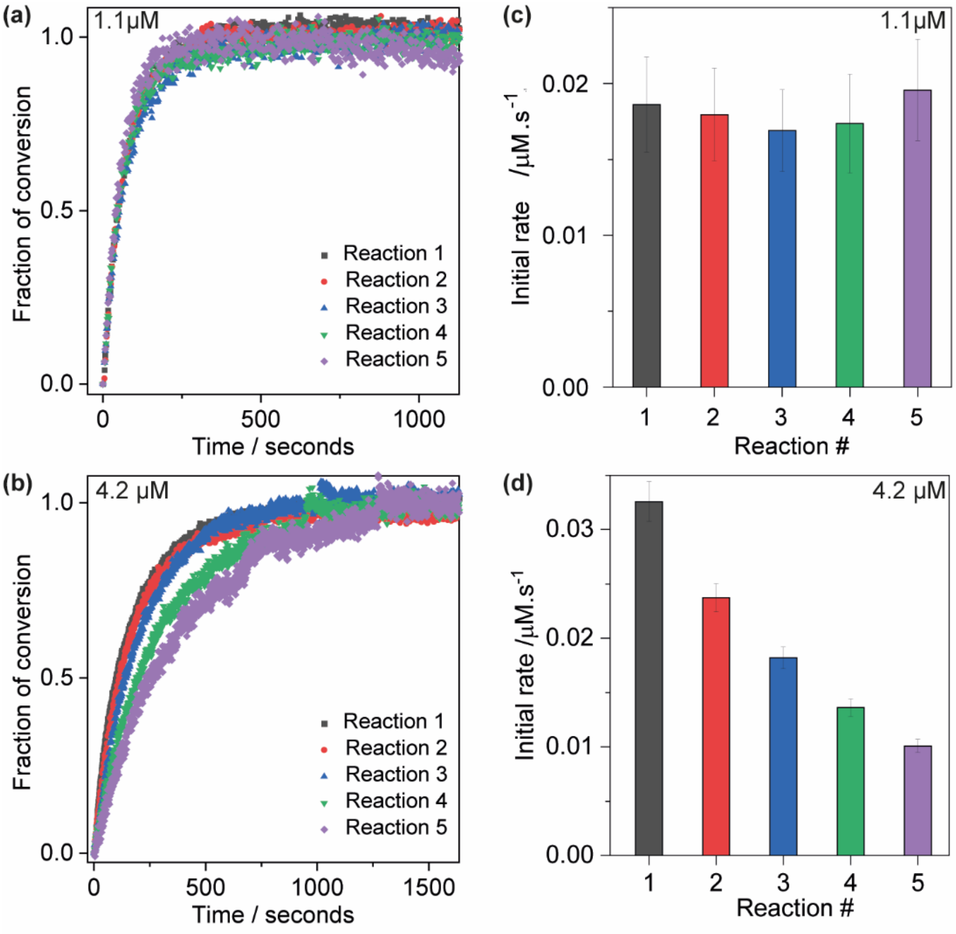

Figure S8. Time-resolved fluorescence measurements for coacervate-based emulsions (10 mg/mL) containing chymotrypsin (5 µg/mL) and copolymer (0.4 mg/mL) in toluene, following five successive additions of substrate at concentrations of **(a)** 1.5 µM and **(b)** 4.2 µM. Each curve corresponds to a separate substrate addition: 1st (■), 2nd (●), 3rd (▲), 4th (▼), and 5th (♦). The corresponding initial reaction rates extracted from each addition are shown in **(c)** and **(d)**, respectively.

For reactions with 1.1 µM substrate (Figure S8c), the initial rates were consistent across all five measurements, with no statistically significant differences detected. The variations observed were within the experimental error. For reactions with 4.2 µM substrate (Figure S8d), the initial rates decreased progressively across the five measurements, showing a clear downward trend from reaction 1 to 5.

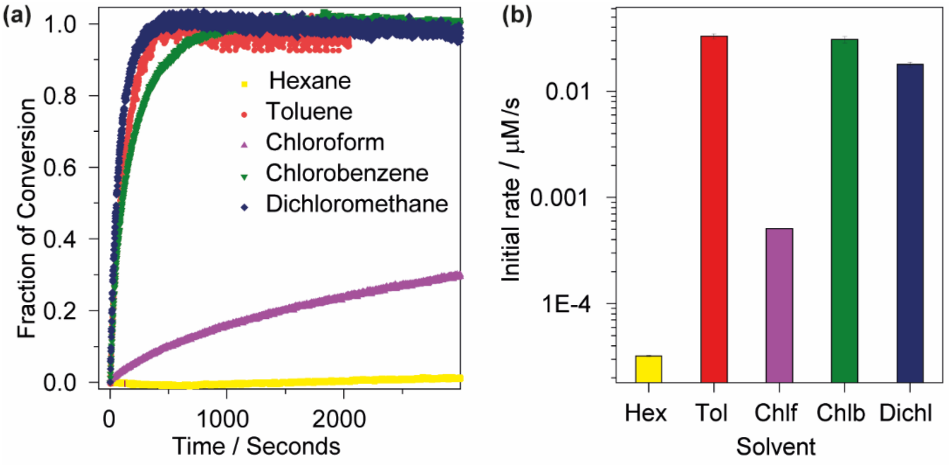

Figure S9. (a) Time-resolved fluorescence measurements for coacervate-based emulsions (10 mg/mL) containing chymotrypsin (5 µg/mL) and copolymer (0.4 mg/mL) in different organic solvents: (■) hexane, (●) toluene, (▲) chloroform, (▼) chlorobenzene, and (♦) dichloromethane after addition of substrate at concentrations of 1.5 µM. **(b)** The corresponding initial reaction rates extracted from each addition.

As shown in Figure S9, enzymatic activity was observed for coacervate-based emulsions in all organic solvents tested, highlighting the broad applicability of this encapsulation protocol. Among the systems evaluated, the emulsion in hexane displayed the lowest initial reaction rate. This behavior arises from the low solubility of the substrate in hexane. Upon addition, the substrate precipitated in the solvent phase, leading to a markedly reduced initial reaction rate. We hypothesize that, in this case, the reaction was limited by both diffusion of the substrate into the coacervate phase and solubility: the substrate would dissolve, be consumed by the enzyme, and only then would additional material solubilize. The substrate may have significantly higher solubility in the coacervate phase; however, this process would also be subject to transport limitations, and it is likely that the substrate is consumed faster than it can diffuse. Thus, for hexane, we hypothesize that the observed initial rate is dominated by mass transfer limitations that are made worse by the poor solubility of the substrate in the organic solvent.

In aqueous coacervate systems, previous studies have shown that reaction rates can increase due to preferential partitioning of the substrate into the coacervate, effectively raising its local concentration.[13–15] We expect that a similar effect may occur in organic-solvent coacervate systems when the substrate strongly partitions into the coacervate phase, which would increase its effective initial concentration and consequently the reaction rate. However, a systematic investigation of substrate partitioning and solubility effects would require experiments using multiple substrates with different

physicochemical properties, which we plan to explore in future work. This analysis is beyond the scope of the current study.

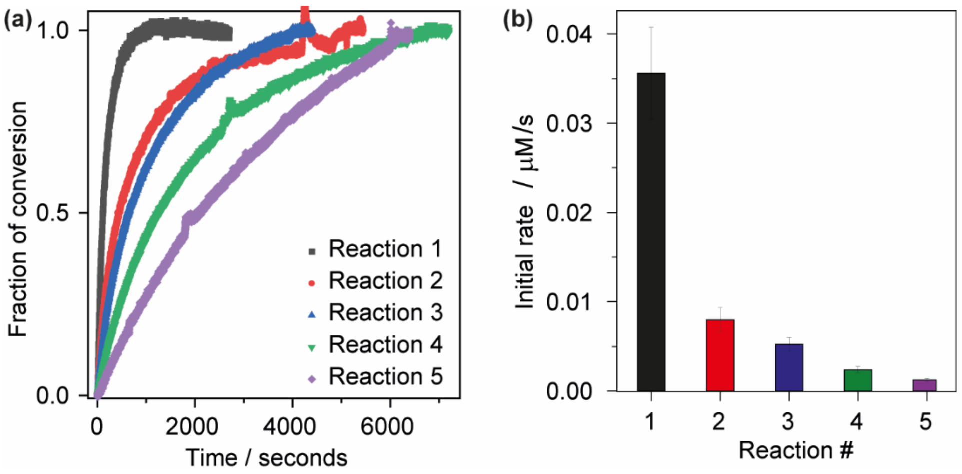

Figure S10. (a) Fluorescence measurements over time for coacervate-based emulsions (10 mg/mL) containing chymotrypsin (5 μg/mL) and copolymer (0.4 mg/mL) in chlorobenzene following substrate addition over five cycles using the same coacervate droplets. Enzymatic activity was evaluated after (■) 1, (●) 2, (▲) 3, (▼) 4, and (♦) 5 cycles of reactions, demonstrating the efficient reuse without significant loss of enzymatic activity. **(b)** The corresponding initial reaction rates extracted from each cycle.

As shown in Figure S10, we observed that the droplets can be reused; however, since the densities of the coacervate phase (1.09 g/cm^3) and chlorobenzene (1.11 g/cm^3) are very close, separating the droplets from the organic solvent requires extensive centrifugation. Therefore, we attribute the slight decrease in reaction rate across cycles to the removal of enzyme-containing coacervate droplets along with the solvent, rather than to an intrinsic loss of enzymatic activity.

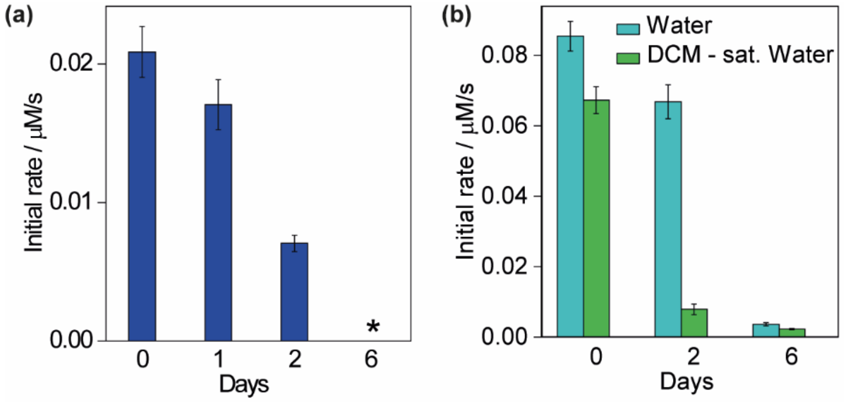

Figure S11. Initial reaction rates of **(a)** coacervate (10 mg/mL) with α-chymotrypsin (5 µg/mL) and copolymer (0.4 mg/mL in DCM over time (days 0, 1, 2 and 6) and **(b)** α-chymotrypsin (5 µg/mL) in water and water saturated with DCM over time (days 0, 2, 6) at pH 8.9. Day 0 corresponds to the measurement performed immediately after sample preparation.

In contrast to the coacervate-based emulsions in toluene and chlorobenzene (Figure S6), enzyme stability over time was markedly reduced in dichloromethane (Figure S11a). Although initial reaction rate for day 0 was similar to that in toluene, prolonged incubation revealed a statistically significant, time-dependent decrease in enzymatic activity, reaching a minimum after six days (Figure S11a). We hypothesize that this loss of activity is due to slow inactivation of the enzyme because of the presence of low levels of dichloromethane in the coacervate phase. Dichloromethane has a significantly higher solubility in water (17.5 mg/mL) than toluene (0.5 mg/mL), thus allowing greater amounts to diffuse into the coacervate phase and potentially compromise enzyme structure and function. A similar trend was observed when α-chymotrypsin was incubated in water saturated with dichloromethane (Figure S11b), where the decline in activity was more pronounced than in pure aqueous buffer. These findings underscore the critical role of solvent selection in preserving long-term enzymatic functionality within coacervate droplets. An interesting open question, however, is why dichloromethane does not inactivate the enzyme more rapidly inside the coacervate droplets, given its small molecular size and ability to diffuse quickly. While this remains beyond the scope of the current work, it highlights an intriguing direction for future investigation.

Effect of Water-Saturated Organic Solvents on Coacervate Dispersion

Highly water-miscible solvents such as diethyl ether, ethyl acetate, and ethanol caused rapid solidification of the coacervate phase due to water extraction from the droplets. To further evaluate how the hydration level of the continuous organic phase affects dispersion, we examined the behavior of coacervates in water-saturated organic solvents.

Diethyl ether and ethyl acetate were saturated with water prior to use, and the copolymer was dissolved in each solvent following the conditions used throughout this study (0.4 mg/mL copolymer; 10 mg/mL coacervate). After addition of the coacervate phase, the mixtures were subjected to vigorous vortexing and bath sonication. In both water-saturated solvents, the coacervate persisted as a single bulk phase and did not fragment into droplets, indicating that increased water content in the continuous phase inhibits droplet formation. The same behavior was observed when the coacervate was dispersed in the water-saturated solvents without copolymer. In contrast, when the copolymer was dissolved in dry diethyl ether or ethyl acetate, the coacervate successfully dispersed throughout the system. Although the coacervate appeared as dehydrated fibers due to water loss, it nonetheless broke apart and distributed within the organic phase, consistent with copolymer-mediated interfacial stabilization.

These results suggest that the presence of additional water in the continuous phase alters the interfacial behavior of the copolymer. A plausible explanation is that water increases the solubility of the copolymer in the bulk solvent, reducing its preferential localization at the coacervate-solvent interface and thereby preventing droplet stabilization.

Partition Coefficient and Product Distribution Analysis

The partition coefficient of the reaction product between the coacervate phase and the surrounding solvent was determined from confocal fluorescence micrographs. Fluorescence intensities were quantified inside and outside the droplets using ImageJ, and the ratio of mean intensities was used to estimate the apparent partition coefficient (K).[16] Fluorescence images were acquired for at least 30 independent droplets across multiple fields of view to enable statistical comparison. From these analyses, the product was found to preferentially localize in the coacervate phase with a partition coefficient of 79 ± 4.

In a system with 10 mg of coacervate dispersed in 1 mL (≈0.010 mL coacervate and 0.990 mL solvent) and an initial substrate concentration of 4.2 μM, mass balance predicts that ~56% of the product resides in the solvent and ~44% in the coacervate at equilibrium. Removing 0.9 mL of the solvent phase (≈91% of the solvent volume) therefore removes ≈51% of the total product in a single rinse. Based on a partition coefficient of 79 and the phase volumes, we further calculate that the product concentration in the droplets should decrease from ~186 μM before rinsing to ~92 μM after one rinse, while the concentration in the bulk solvent decreases from ~2.36 to ~1.17 μM.

Importantly, because the coacervate volume is very small compared to the bulk solvent, a large fraction of the product can still be removed even when the partition coefficient is high (Table S2). This feature underscores the efficiency of the recycling system: despite the strong preferential localization of the product inside the coacervate, the large solvent volume ensures that product removal during solvent exchange captures a significant percentage of the total product.

Table S2. Percentage of product removed after withdrawing 0.9 mL of the solvent phase (out of 0.99 mL total), for different example partition coefficients (K).

Partition coefficient	Fraction of product in solvent (%)	% of total product removed after 0.9 mL solvent removal
1 (example)	~99	~91
10 (example)	~90	~82
79 ± 4 (measured)	~56	~51
100 (example)	~50	~46

Quantification of Copolymer Remaining in the Continuous Phase

To quantify the amount of copolymer remaining in the continuous organic phase after emulsification, we prepared coacervate-in-toluene emulsions (0.8 mg/mL copolymer; 10 mg/mL coacervate), centrifuged the samples to separate the coacervate droplets, and collected 1.0 mL of the supernatant. The toluene was then evaporated, and the residue was dissolved in 0.5 mL of $CDCl_3$ for ^1H NMR analysis. For comparison, a reference solution containing 0.8 mg/mL of copolymer in $CDCl_3$ was also prepared. Figures S12 and S13 show the ^1H NMR spectra of the reference copolymer solution and of the supernatant containing the residual copolymer.

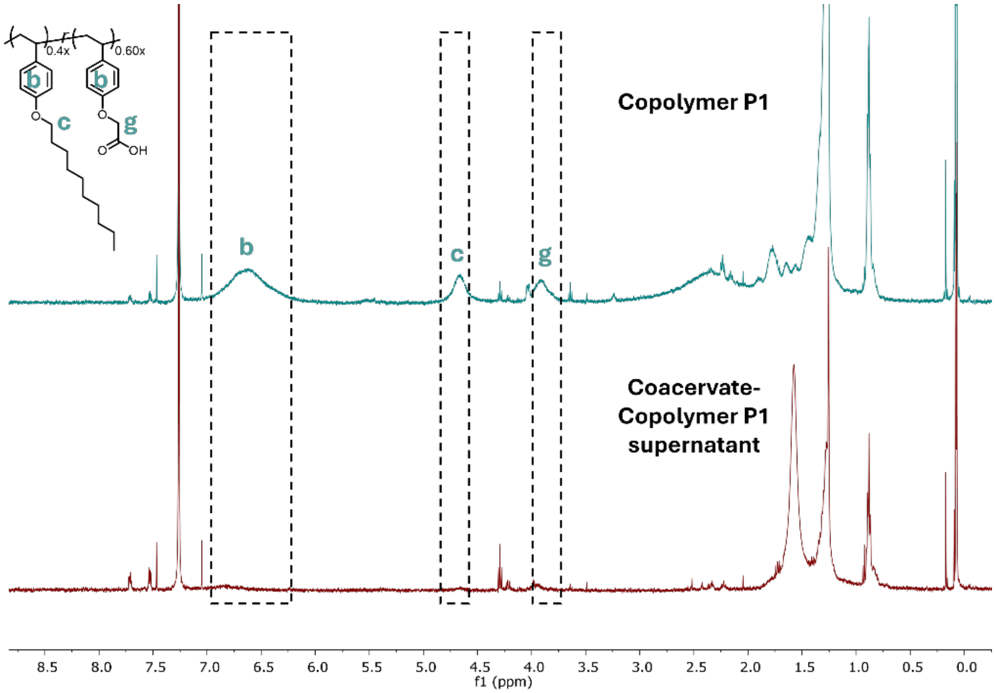

Figure S12. ^1H NMR spectrum (400 MHz, $CDCl_3$) of (---, top) copolymer P1 solution in $CDCl_3$ (0.8 mg/mL) and (---, bottom) copolymer P1 in the supernatant.

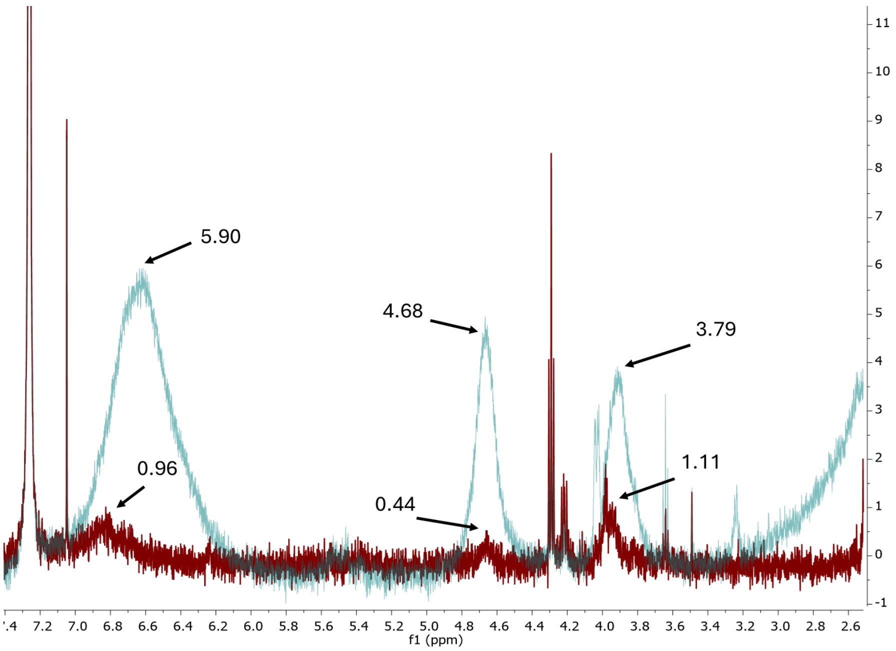

Figure S13. Zoomed in image of ^{1}H NMR spectrum (400 MHz, $CDCl_3$) of (---) copolymer P1 solution in $CDCl_3$ (0.8 mg/mL) and (---) copolymer P1 in the supernatant. Numbers indicate peak intensities of corresponding monomer backbone and side chain.

The relative peak intensities at characteristic copolymer resonances were used to estimate the percentage of copolymer remaining in the organic phase. Using the peak intensities from three representative peaks (peaks b, c, and g), the percentage of copolymer loss to the continuous phase was calculated as:

- Peak b (PS backbone of both monomers): 0.96 / 5.90 × 100 = 16.7%
- Peak c (monomer 1): 0.44 / 4.68 × 100 = 9.4%
- Peak g (monomer 2):1.11 / 3.79 × 100 = 29.3%

To compare to peak b, which represents both monomers, peaks c and g were combined according to their relative ratios: Combining the contributions from peaks c and g according to their relative ratios: (0.4 × 9.4%) + (0.6 × 29.3%) = ~21% loss. Because the supernatant was concentrated two-fold during sample preparation (1.0 mL of toluene reduced to 0.5 mL of $CDCl_3$), all values were corrected by dividing by two. After applying this correction, the results indicate that only ~8-10% of the initial copolymer remained truly dissolved in the continuous phase, confirming that the vast majority partitions to the coacervate-toluene interface rather than remaining free in solution. Considering the excess of volume of organic phase with respect to coacervate (100:1), these results suggest that the copolymer strongly partitions to the coacervate/organic interface, and that the amount of free copolymer in the continuous phase is small.

Characterization of the Copolymers

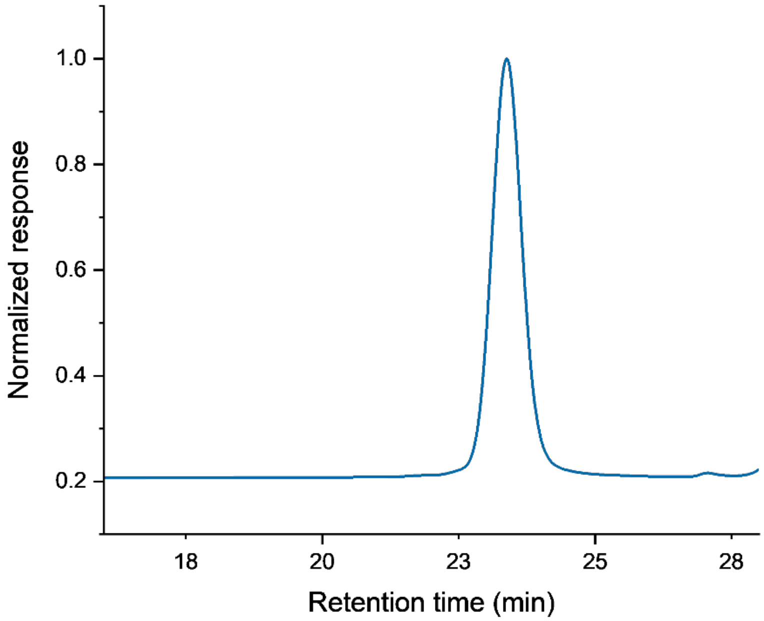

Figure S14. GPC trace of polymer **P0** shows the presence of a polymer with M_w: 9.6 kDa, M_n: 9.3 kDa, and Đ: 1.03. GPC was performed with a refractive index detector (ambient temperature, flow rate 1 mL/min), tetrahydrofuran (THF) as the eluent, and analysis was calibrated using a polystyrene standard and 1 µL of toluene was added as the internal reference.

NMR Spectra:

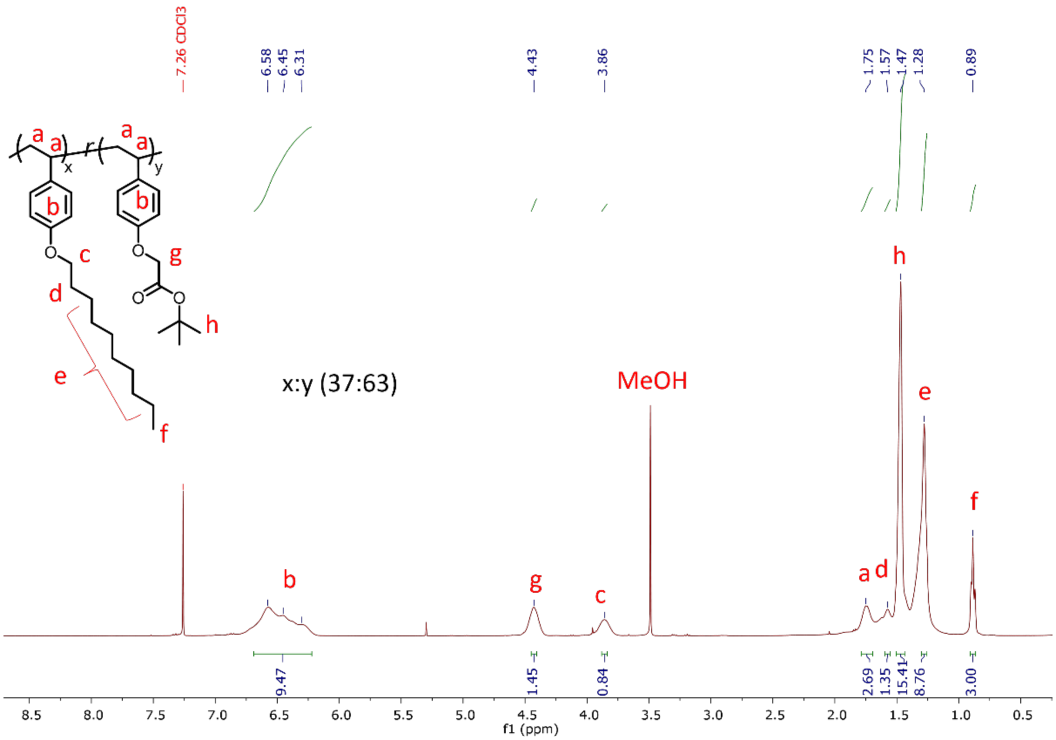

Figure S15. ^{1}H NMR spectrum (400 MHz, $CDCl_3$) of polymer P0. δ (ppm): 6.58, 6.45, 6.31, 4.43, 3.86, 1.75, 1.57, 1.47, 1.28, 0.89. Integration of the methylene proton adjacent to the phenol group in both the alkyl unit (c) and the carboxylate unit (g) was used to determine the molar ratio of monomers (decyl/carboxylate = 4:6).

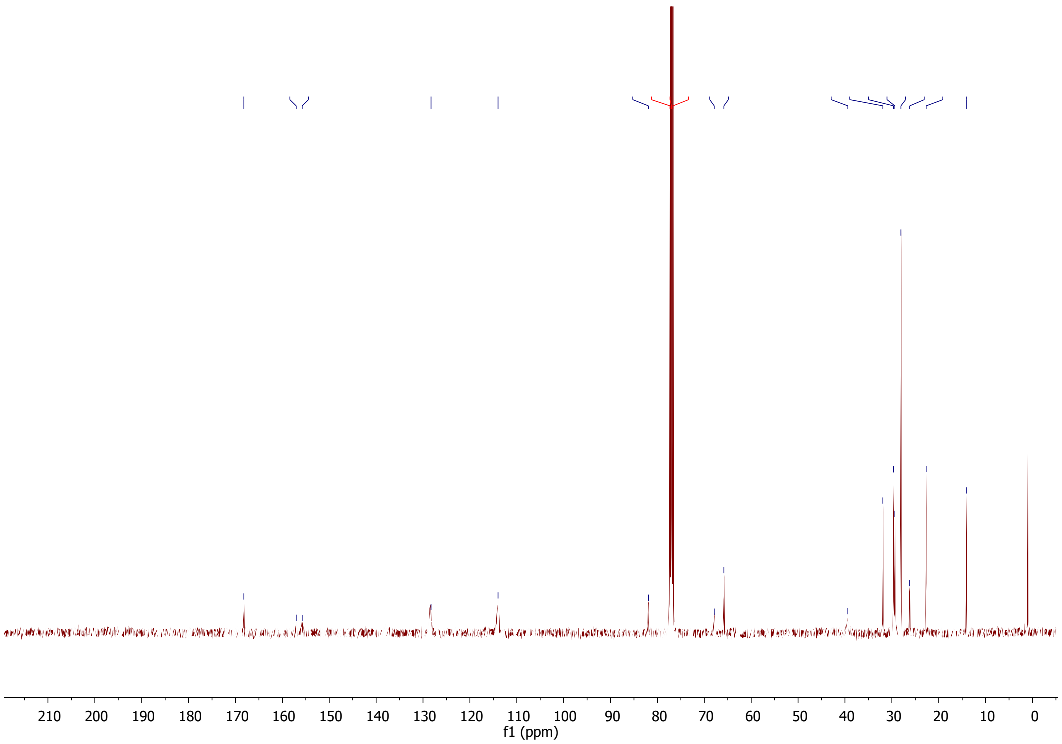

Figure S16. ^{13}C NMR spectrum (100 MHz, $CDCl_3$) of polymer P0. δ (ppm): 155.52, 155.50, 128.35, 114.33, 67.93, 65.44, 65.02, 39.42, 31.91, 29.61, 29.35, 26.13, 22.69, 14.13.

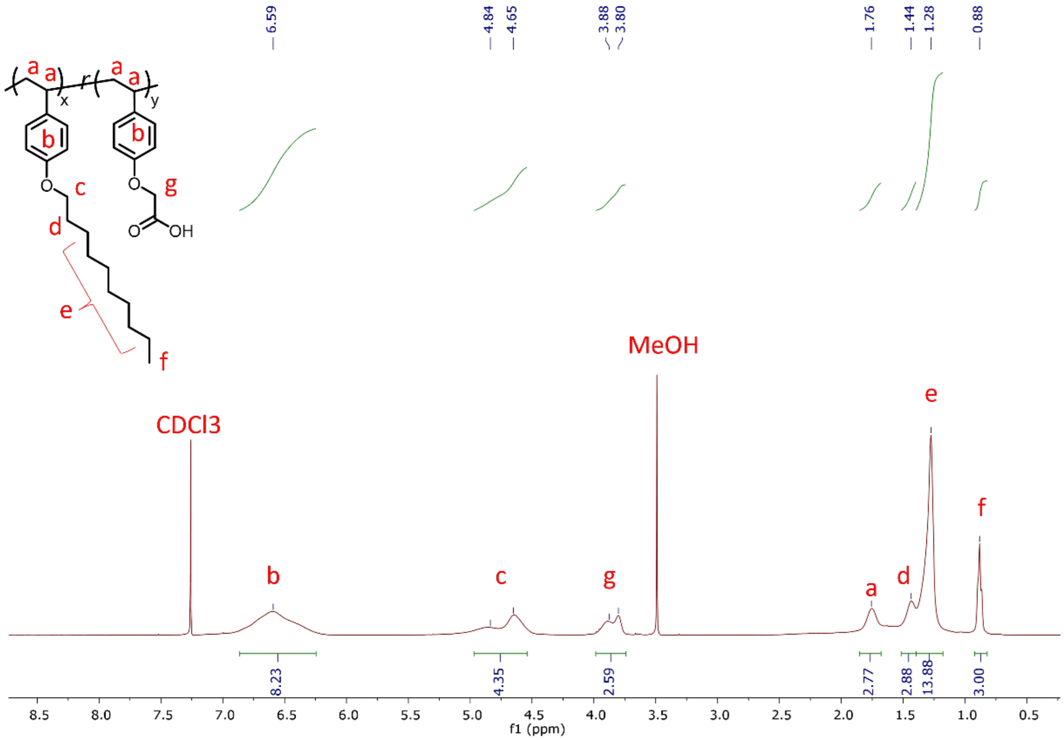

Figure S17. ^{1}H NMR spectrum (400 MHz, $CDCl_3$) of polymer P1. δ (ppm): 6.59, 4.85, 4.65, 3.88, 3.80, 3.49, 1.76, 1.44, 1.28, 0.88. The sharp decrease in integration at δ 1.44 indicates successful tert-butyl group deprotection. Integration of the methylene proton signals in alkyl (c) and carboxylate (g) units confirmed the molar ratio of monomers (decyl/carboxylate = 4:6).

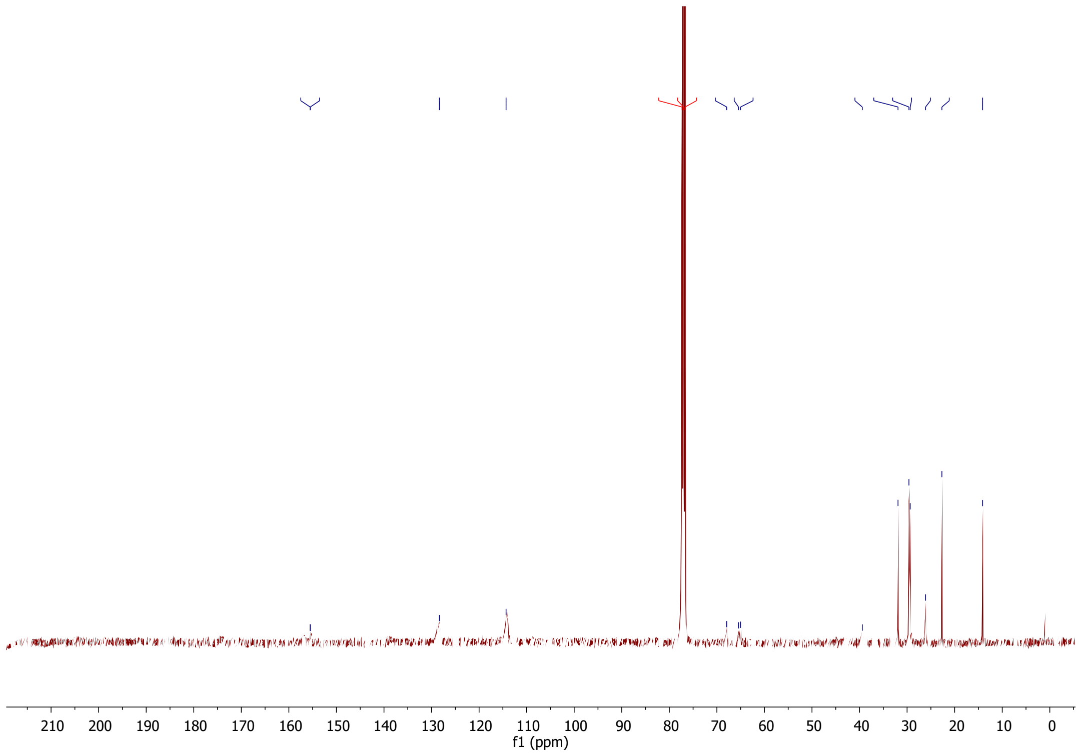

Figure S18. ^{13}C NMR spectrum (100 MHz, $CDCl_3$) of polymer P1. δ (ppm): 155.52, 155.50, 128.35, 114.33, 67.93, 65.44, 65.02, 39.42, 31.91, 29.61, 29.35, 26.13, 22.69, 14.13.

References

[1] B. Zhao, M. A. C. Serrano, J. Gao, J. Zhuang, R. W. Vachet, S. Thayumanavan, "Self-assembly of random co-polymers for selective binding and detection of peptides" *Polym. Chem.* **2018**, *9*, 1066–1071.

[2] J. Gao, B. Zhao, M. Wang, M. A. C. Serrano, J. Zhuang, M. Ray, V. M. Rotello, R. W. Vachet, S. Thayumanavan, "Supramolecular Assemblies for Transporting Proteins Across an Immiscible Solvent Interface" *J. Am. Chem. Soc.* **2018**, *140*, 2421–2425.

[3] A. Svensson, L. Piculell, B. Cabane, P. Ilekti, "A new approach to the phase behavior of oppositely charged polymers and surfactants" *J. Phys. Chem. B* **2002**, *106*, 1013–1018.

[4] A. Svensson, J. Norrman, L. Piculell, "Phase Behavior of Polyion−Surfactant Ion Complex Salts: Effects of Surfactant Chain Length and Polyion Length" *J. Phys. Chem. B* **2006**, *110*, 10332–10340.

[5] N. Ui, "Isoelectric points and conformation of proteins" *Biochim. Biophys. Acta - Protein Struct.* **1971**, *229*, 582–589.

[6] N. Woitovich Valetti, J. Lombardi, V. Boeris, G. Picó, "Precipitation of chymotrypsin from fresh bovine pancreas using ι-carrageenan" *Process Biochem.* **2012**, *47*, 2570–2574.

[7] M. Gholizadeh, B. Shareghi, S. Farhadian, "Revealing the interaction between alpha-chymotrypsin and eugenol: An integrated multi-spectral and dynamic simulation approach" *Int. J. Biol. Macromol.* **2024**, *277*, 134504.

[8] W. C. Blocher McTigue, S. L. Perry, "Design rules for encapsulating proteins into complex coacervates" *Soft Matter* **2019**, *15*, 3089–3103.

[9] W. C. Blocher McTigue, S. L. Perry, "Design rules for encapsulating proteins into complex coacervates" *Soft Matter* **2019**, *15*, 3089–3103.

[10] M. Spitzer, E. Sabadini, W. Loh, "Poly(ethylene glycol) or Poly(ethylene oxide)?: Magnitude of end-group Contribution to the Partitioning of Ethylene Oxide Oligomers and Polymers between Water and Organic Phases" *J. Braz. Chem. Soc.* **2002**, *13*, 7–9.

[11] S. Gao, S. Srivastava, "Comb Polyelectrolytes Stabilize Complex Coacervate Microdroplet Dispersions" *ACS Macro Lett.* **2022**, *11*, 902–909.

[12] C. Fick, Z. Khan, S. Srivastava, "Interfacial stabilization of aqueous two-phase systems: a review" *Mater. Adv.* **2023**, *4*, 4665–4678.

[13] C. A. Strulson, R. C. Molden, C. D. Keating, P. C. Bevilacqua, "RNA catalysis through compartmentalization" *Nat. Chem.* **2012**, *4*, 941–946.

[14] I. B. A. Smokers, B. S. Visser, A. D. Slootbeek, W. T. S. Huck, E. Spruijt, "How Droplets Can Accelerate Reactions─Coacervate Protocells as Catalytic Microcompartments" *Acc. Chem. Res.* **2024**, *57*, 1885–1895.

[15] D. Q. P. Reis, S. Pereira, A. P. Ramos, P. M. Pereira, L. Morgado, J. Calvário, A. O. Henriques, M. Serrano, A. S. Pina, "Catalytic peptide-based coacervates for enhanced function through structural organization and substrate specificity" *Nat. Commun.* **2024**, *15*, 9368.

[16] J. Lee, F. Pir Cakmak, R. Booth, C. D. Keating, "Hybrid Protocells Based on Coacervate-Templated Fatty Acid Vesicles Combine Improved Membrane Stability with Functional Interior Protocytoplasm" *Small* **2024**, *20*, 2406671.
